# The Relationship between Gene Network Structure and Expression Variation among Individuals and Species

**DOI:** 10.1371/journal.pgen.1005398

**Published:** 2015-08-28

**Authors:** Karen E. Sears, Jennifer A. Maier, Marcelo Rivas-Astroza, Rachel Poe, Sheng Zhong, Kari Kosog, Jonathan D. Marcot, Richard R. Behringer, Chris J. Cretekos, John J. Rasweiler, Zoi Rapti

**Affiliations:** 1 School of Integrative Biology, University of Illinois, Urbana, Illinois, United States of America; 2 Institute for Genomic Biology, University of Illinois, Urbana, Illinois, United States of America; 3 Department of Bioengineering, University of California, San Diego, La Jolla, California, United States of America; 4 Department of Mathematics, University of Illinois, Urbana, Illinois, United States of America; 5 Department of Genetics, University of Texas MD Anderson Cancer Center, Houston, Texas, United States of America; 6 Department of Biological Sciences, Idaho State University, Pocatello, Idaho, United States of America; 7 Department of Obstetrics and Gynecology, State University of New York Downstate Medical Center, Brooklyn, New York, United States of America; University of California Davis, UNITED STATES

## Abstract

Variation among individuals is a prerequisite of evolution by natural selection. As such, identifying the origins of variation is a fundamental goal of biology. We investigated the link between gene interactions and variation in gene expression among individuals and species using the mammalian limb as a model system. We first built interaction networks for key genes regulating early (outgrowth; E9.5–11) and late (expansion and elongation; E11-13) limb development in mouse. This resulted in an Early (ESN) and Late (LSN) Stage Network. Computational perturbations of these networks suggest that the ESN is more robust. We then quantified levels of the same key genes among mouse individuals and found that they vary less at earlier limb stages and that variation in gene expression is heritable. Finally, we quantified variation in gene expression levels among four mammals with divergent limbs (bat, opossum, mouse and pig) and found that levels vary less among species at earlier limb stages. We also found that variation in gene expression levels among individuals and species are correlated for earlier and later limb development. In conclusion, results are consistent with the robustness of the ESN buffering among-individual variation in gene expression levels early in mammalian limb development, and constraining the evolution of early limb development among mammalian species.

## Author Summary

The variation generating mechanisms of development interact with the variation sorting mechanism of natural selection to produce organismal diversity. While the impacts of natural selection on existing variation have received much study, those of development on the generation of this variation remain less understood. This fundamental gap in our knowledge restricts our understanding of the key processes shaping evolution. In this study, we combine mathematical modeling, and population-level and cross-species assays of gene expression to investigate the relationship between the structure of the gene interactions regulating limb development and variation in the expression of limb genes among individuals and species. Results suggest that the way in which genes interact (i.e., development) biases the distribution of variation in gene expression among individuals, and that this in turn biases the distribution of variation among species.

## Introduction

Phenotypic variation within populations is a prerequisite of evolution by natural selection, and in theory has the potential to bias the trajectory and rate of evolutionary change [1–6]. As such, identifying the processes that shape phenotypic variation has long been a fundamental pursuit of evolutionary biologists. Historically, evolutionary biologists have tended to focus on the sorting of population-level variation by selective processes, rather than on the production of that variation by developmental processes [7]. As a result, the effect of developmental processes on the distribution and magnitude of phenotypic variation among individuals and species remains unclear for most systems. In this study we use the mammalian limb as a study system to investigate two questions that address the relationship between developmental processes and phenotypic variation at the level of gene expression dynamics: (1) Does the structure of the gene network affect the distribution of variation in gene expression among individuals?, and (2) Is the distribution of variation in gene expression among individuals correlated with the evolutionary divergence in gene expression among species?

**Fig 1:**
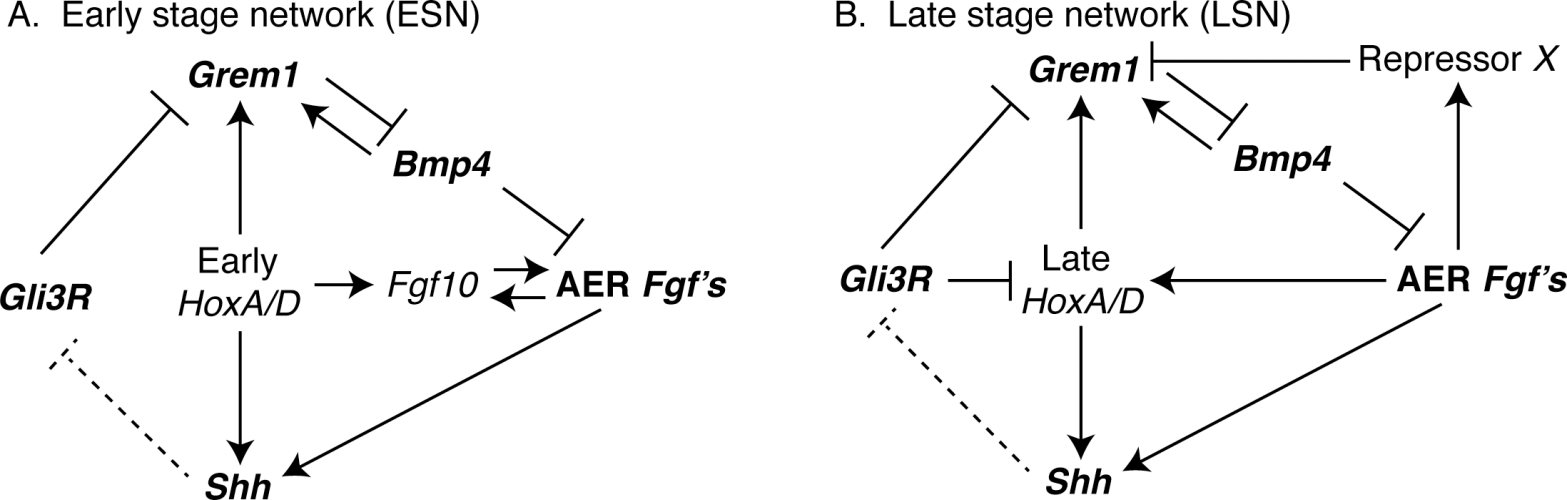
Interactions among genes in the (A) Early (ESN) and (B) Late (LSN) stage networks that were computationally modeled in this study are shown. Networks were based on Bénazet et al. (2009) and Sheth et al. (2013). Note that some aspects of the ESN and LSN differ.

The mammalian limb is an ideal system for examining these questions because its development is well characterized, its morphology diverse, and since its form is central to many mammalian behaviors, its morphology is certainly under selection [8–12]. Many of the critical gene interactions that regulate limb outgrowth and patterning in mouse, the traditional mammal model, have been identified [9,10,13]. Initial budding of the limb from the body and limb outgrowth (embryonic day [E] 9.5 –E11) are regulated by interactions between several genes, including *Bmp4*, *Gli3*, *Grem1*, *Shh*, AER-*Fgf*’s (e.g., *Fgf4*, *Fgf8*), *Fgf10*, and *Hox* genes (Fig 1A). Knockouts of these genes result in pathological phenotypes ranging from severe (e.g., complete limb agenesis; AER-*Fgf*’s, *Fgf10*) to moderate (e.g., limb truncations; *Bmp4*) to mild (e.g., malformed digits; *Shh*, *Gli3*, *Grem1*) [13–18]. Most of these genes (e.g., *Bmp4*, *Gli3*, *Grem1*, *Shh*, AER-*Fgf*’s, and *Hox* genes) are also involved in later limb outgrowth and patterning (E11 –E13), but some of their interactions differ (e.g., *Hox* genes and *Gli3*, *Shh*, AER-*Fgf*’s; Fig 1B). As a result, the structure of the gene regulatory network differs for earlier (E9.5 –E11) and later (E11 –E13) limb development. This structural difference provides two opportunities to investigate the relationship between network structure and gene expression variation among individuals.

This structural difference also provides an opportunity to contrast earlier and later limb development. Research suggests that the main segments of the limb (e.g., stylopod, zeugopod, and autopod) are specified by or during the time of initial limb outgrowth [19,20]. As a result, disruption of early limb development could have potentially catastrophic effects on limb formation that are not likely to be selectively advantageous (e.g., limb agenesis). In contrast, disruptions of later limb development are less likely to have as severe an impact on the overall limb structure. While later disruptions might impact the relative size of limb segments, they are less likely to result in no limb at all. Following this logic, we might hypothesize that genes regulating early limb development generally exhibit less variation in expression among individuals than those regulating later limb development [21–33]. Additionally, it is possible that select early limb genes might vary at a level equal to or greater than that of individual later genes, but that this variation is dampened at the system level by the interactions among genes that characterize the gene network (i.e., developmental buffering) [34–37]. As population-level variation provides the raw material upon which natural selection acts, we can further hypothesize that the genes regulating early limb development also exhibit less variation in expression among species [38]. Support for these hypotheses would reinforce the importance of network structure (i.e., development) in shaping variation in mammalian limbs among individuals and over evolutionary time, while failure to support these hypotheses would suggest that network structure does not play a critical role in the generation of limb variation.

To test these hypotheses, we computationally modeled the gene networks regulating mouse limb development, and determined the sensitivity of network genes to system perturbation and the ability of network genes to perturb the system when altered. We also assessed the sensitivity of the system as a whole to perturbations in gene interactions and expression. We experimentally quantified naturally occurring variation in the expression of several network genes within a population of mouse individuals. To compare variation among species, we used transcriptomic data (RNASeq) from four mammals with divergent limb morphologies (bat, opossum, pig and mouse). We then assessed the relationship between gene and network sensitivity and gene expression variation among mouse individuals and among mammalian species. Our results suggest that the gene network that regulates early limb development is more robust than that regulating later limb development, and that this robustness buffers variation in early limb gene expression among individuals, and constrains the evolution of early limb development among species.

## Results

### Model construction (computational)

We assembled early (ESN) and late (LSN) stage networks for key genes regulating limb development from previously published experimental studies [9,13] (Fig 1). The ESN regulates initial limb outgrowth and the initiation of the epithelial-mesenchymal interactions that are critical to continued limb development. These events occur from embryonic days (E) 9.5 to E11 in mouse. The LSN, in contrast, regulates the limb’s differentiation along its anterior-posterior (i.e., thumb to pinky) axis and elongation along its proximal-distal (shoulder to fingertips) axis from E11 to E13 in mouse. To describe the temporal behavior of the activity (i.e., expression) levels of genes in these networks we built mathematical models (see S1 Methods).

### Model simulations (computational)

After building the models, we ran a series of simulations in which we computationally interrupted interactions between genes and compared the resulting expression levels with those of the unaltered, default model (Table 1).

**Table 1:** Effects of removals of gene-to-gene interactions in simulations on gene expression level. Asterisks indicate values that differ more than 10% from values generated by the unaltered model. The total number of values that differ more than 10% are shown in the last column and row. In total, 14 of 77 (18%) possible expression levels are affected by alterations in the ESN, while 52 of 84 (62%) possible expression levels are affected by alterations in the LSN.

**ESN**	Aer-*Fgf*'s	*Bmp4*	*Fgf10*	*Gli3R*	*Grem1*	*HoxA/D*	*Shh*	Rep *X*	SUM
Unaltered model	1.979	0.011	1.976	0.013	1.988	1.798	1.973	N/A	
Aer-Fgf’s to Fgf10	1.926	0.011	0.989*	0.013	1.988	1.798	1.972	N/A	1
Aer-Fgf’s to Shh	1.979	0.012	1.976	0.068*	1.981	1.798	0.990*	N/A	2
Bmp4 to Aer-Fgf’s	0.989*	0.011	1.921	0.014	1.988	1.798	1.897	N/A	1
Bmp4 to Grem1	1.975	0.013	1.975	0.013	1.977	1.798	1.972	N/A	0
Fgf10 to Aer-Fgf’s	0.990*	0.012	1.908	0.016	1.986	1.798	1.882	N/A	1
Gli3R to Grem1	1.976	0.058*	1.976	0.013	1.006*	1.798	1.973	N/A	2
Grem1 to Bmp4	1.982	0.003*	1.976	0.013	1.985	1.798	1.973	N/A	1
Hox A/D to Grem1	1.972	0.058*	1.975	0.013	1.016*	1.798	1.972	N/A	2
Hox A/D to Shh	1.979	0.012	1.976	0.074*	1.969	1.798	0.983*	N/A	2
HoxA/D to Fgf10	1.923	0.011	0.988*	0.013	1.988	1.798	1.971	N/A	1
Shh to Gli3R	1.979	0.011	1.976	0.003*	1.991	1.798	1.973	N/A	1
SUM	2	3	2	3	2	0	2	N/A	14/77
**LSN**									
Unaltered model	0.791	0.919	N/A	0.010	0.307	1.946	1.767	0.052	
Aer-Fgf’s to Hox A/D	0.599*	1.056*	N/A	0.017*	0.273*	1.000*	1.431*	0.052	6
Aer-Fgf’s to Repressor X	2.000*	0.018*	N/A	0.009	1.910*	1.992	1.925	0.000*	4
Aer-Fgf’s to Shh	0.787	0.922	N/A	0.068*	0.306	1.930	0.957*	0.052	2
Bmp4 to Aer-Fgf’s	0.000*	0.400*	N/A	1.000*	0.635*	0.060*	0.006*	0.000*	7
Bmp4 to Grem1	0.054*	1.989*	N/A	1.000*	0.072*	0.063*	0.001*	0.000*	7
Gli3R to Grem1	0.222*	1.477*	N/A	0.029*	0.208*	1.400*	1.112*	0.049	6
Gli3R to Hox A/D	0.597*	1.063*	N/A	0.017*	0.267*	0.892*	1.397*	0.052	6
Grem1 to Bmp4	2.000*	0.000*	N/A	0.009	0.192*	1.992	1.925	0.054	3
Hox A/D to Grem1	0.430*	1.210*	N/A	0.014*	0.240*	1.776	1.453*	0.052	5
HoxA/D to Shh	0.798	0.916	N/A	0.083*	0.307	1.940	0.813*	0.052	2
Repressor X to Grem1	2.000*	0.018*	N/A	0.009	1.910*	1.992	1.925	0.052	3
Shh to Gli3R	0.721	0.966	N/A	0.000*	0.295	1.932	1.730	0.053	1
SUM	9	9	N/A	9	9	5	8	3	52/84

Within the ESN, removal of the *Hox* to *Grem1* or *Gli3R* to *Grem1* link affects *Grem1* and *Bmp4* expression levels (i.e., alters expression by 10% or more), but does not affect the expression levels of other genes. Removal of the AER-*Fgf*’s to *Shh* or *Hox* to *Shh* links only affects the expression levels of *Gli3R* and *Shh*, removal of the *Fgf10* to AER-*Fgf*’s or *Bmp4* to AER-*Fgf*’s links only affects the expression level of the AER-*Fgf*’s, and removal of the *Hox* to *Fgf10* or AER-*Fgf*’s to *Fgf10* links only affects the expression level of *Fgf10*. Removal of the *Shh* to *Gli3R* link affects the expression level of *Gli3R*, while removal of the *Grem1* to *Bmp4* affects the expression level of *Bmp4*. Removal of the *Bmp4* to *Grem1* link results in no significant change in expression levels. In total, 14 of 77 possible interactions are affected (i.e. expression levels change by 10% or more) by alterations in the ESN (18%) (Table 1).

For the LSN, removal of the *Shh* to *Gli3R* link affects *Gli3R* expression levels, but does not affect the expression levels of other genes. Removal of the AER-*Fgf*’s to *Shh* and *Hox* to *Shh* links affects only *Shh* and *Gli3R* expression levels. Removal of the Repressor *X* to *Grem1* or *Grem1* to *Bmp4* link disrupts the expression levels of *Bmp4*, *Grem1*, and the AER-*Fgf*’s. Removal of the AER-Fgf’s to Repressor *X* link also disrupts the expression levels of *Bmp4*, *Grem1*, and the AER-*Fgf*’s, but also affects Repressor *X* expression levels. Removal of the *Hox* to *Grem1* link affects the expression levels of *Bmp4*, *Gli3R*, *Grem1*, *Shh*, and the AER-*Fgf*’s. Removal of the *Gli3R* to *Hox*, AER-*Fgf*’s to *Hox*, or *Gli3R* to *Grem1* links disrupts the expression levels of all genes save Repressor *X*. Finally, when the *Bmp4* to *Grem1*, *Bmp4* to AER-*Fgf*’s link is removed, expression levels of all genes are affected. In total, 52 of 84 possible interactions are affected (i.e. expression levels change by 10% or more) by alterations in the LSN (62%) (Table 1).

**Fig 2:**
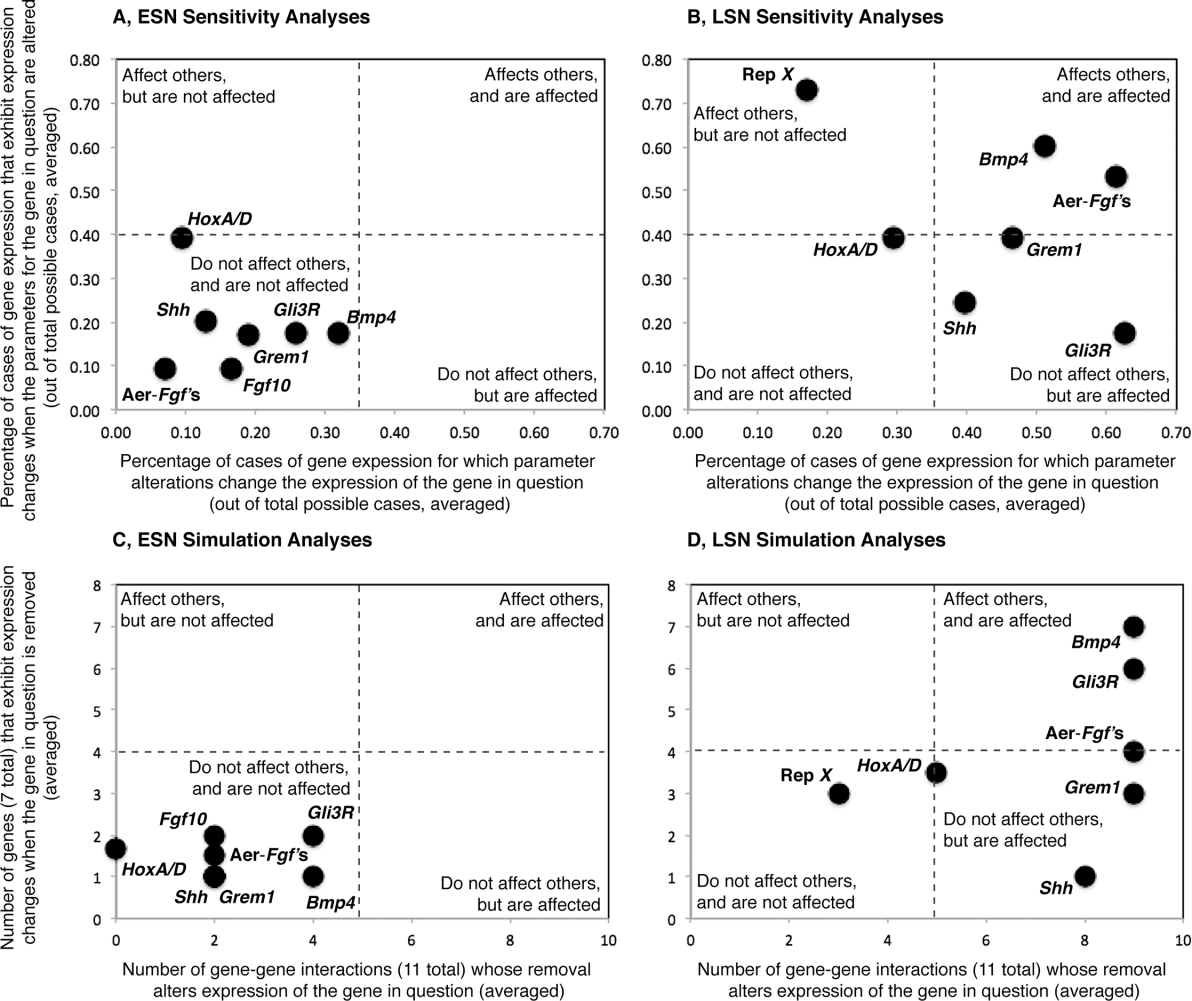
Visualizations of the results of the sensitivity and simulation analyses, all plots are shown at the same scale. The ability of alterations in gene-related parameter values (e.g., p_b_, K_10_, a_b_ for ESN *Bmp4*) to impact gene expression levels (Y-axes) and the sensitivity of gene expression levels to parameter value alterations (X-axes) are shown for the Early (A) and Late (B) stage networks. The ability of individual genes to impact the expression levels of other genes when removed (Y-axes) and the sensitivity of the expression levels of individual genes to the removal of other genes (X-axes) are also shown for the Early (C) and Late (D) stage networks. Both sensitivity (A) and simulation (C) analyses suggest that perturbations in the ESN tend not to have a great effect on gene expression levels, and ESN gene expression levels tend not to be greatly affected by perturbations. Sensitivity (B) and simulation (D) analyses also suggest that perturbations in the LSN have a greater ability to disrupt gene expression values and the expression levels of LSN genes are more sensitive to perturbations than are those of ESN genes.

For each gene in each model, we then determined the number of genes whose removal alters expression of the gene in question (i.e. expression levels change by 10% or more), and the number of genes that exhibit expression changes when the gene in question is removed. We used the resulting values to generate a simulation space that was used to evaluate the ability of genes to affect other genes, and to be affected themselves by network perturbations (Fig 2C and 2D). Simulation spaces for the ESN and LSN were generated using the same scales to allow comparisons. For the ESN (Fig 2C), all genes fall in the lower left quadrant of the space, suggesting that they do not greatly affect expression of other genes, and are not greatly affected by others. In contrast, most LSN genes (Fig 2D) fall in the right upper and lower quadrants of simulation space. Genes in both the upper (e.g., *Bmp4*, *Gli3R*) and lower (e.g., AER-*Fgf*’s, *Grem1*, *Shh*) right quadrants and their boundaries are affected by perturbations in other genes, but genes in the upper right quadrant also affect the expression of other genes while genes in the lower right quadrant do not. *Hox* A/D falls in near the middle of the simulation space, suggesting that it moderately affects others and is affected by them. Only Repressor *X* falls in the lower left quadrant for the LSN, suggesting that it does not affect others and is not affected itself.

### Sensitivity analyses (computational)

We next varied the parameter values used in the models and compared the resulting gene expression levels to those of the unaltered, default model (S1 Table). Results indicate that the ESN is most sensitive to changes in *Hox A/D* parameters (39% of *Hox A/D* parameter changes result in a ≥10% change in the expression level of another gene), followed by *Shh* (20%) and *Bmp4* (18%), *Gli3R* (18%), and *Grem1* (17%). The ESN is less sensitive to changes in AER-*Fgf* (10%) and *Fgf10* (9%) parameters. *Bmp4* is the most sensitive of the ESN genes to changes in parameters of other genes (32% of parameter changes result in a ≥10% change in the expression level of *Bmp4*), followed by *Gli3R* (26%), *Grem1* (19%), *Fgf10* (17%), and *Shh* (13%). *Hox A/D* (9%) and *Fgf8* (7%) expression levels are less sensitive to ESN parameter changes.

The LSN is more sensitive to changes in Repressor *X* (73%), *Bmp4* (60%), and the AER-*Fgf*’s (53%), and less sensitive to changes in *Hox A/D* (39%), *Grem1* (39%), *Shh* (25%) and *Gli3R* (18%). Within the LSN, the AER-*Fgf*’s (61%) and *Gli3R* (63%) are the most sensitive to parameter changes in other genes, followed by *Bmp4* (51%), *Grem1* (47%), and *Shh* (40%), while *Hox A/D* (30%) and Repressor *X* (17%) are less sensitive.

The percentages listed above were used to generate a sensitivity space, similar to the simulation space described above (Fig 2A and 2B). ESN and LSN sensitivity spaces were generated using the same scales to facilitate comparisons. Similar to the simulation results, all ESN genes group within or on the boundary of the lower left quadrant of the space, suggesting that alteration of the values of their related-parameters does not greatly affect expression of other genes, and that their expression levels are not greatly affected by alterations in the values of the related-parameters of other genes. LSN genes are more distributed in the sensitivity space. *Bmp4* and the AER-*Fgf*’s lie in the upper right quadrant, similar to their location in the simulation space (Fig 2C). *Grem1*, *Shh*, *Gli3R* and fall in or on the boundary of the lower right quadrant, indicating that they do not affect others but are affected themselves. Of these, *Grem1* and *Shh* also fall within the lower right quadrant of the simulation space (Fig 2C). Repressor *X* lies within the upper left quadrant, suggesting that alteration of the values of its related-parameters affects the expression of other genes but that its expression is not greatly affected alterations in the values of the related-parameters of other genes. Repressor *X* also falls on the left side of the simulation space, but in the lower quadrant. Similar to its location in the simulation space, *Hox A/D* falls near the center of the plot.

### Among individual variation in gene expression (qPCR)

We performed a series of real-time quantitative PCR (qPCR) assays to quantify the expression levels of genes that appear in both the ESN and LSN models (*Bmp4*, *Gli3*, *Grem1*, *Shh*, and the AER-*Fgf Fgf8*) in mouse embryos. For the early developmental stages (ES), the averaged, scaled expression level was 2.02 for *Bmp4*, 2.58 for *Fgf8*, 2.19 for *Grem1*, 1.68 for *Shh*, and 0.02 for *Gli3*. For the later developmental stages (LS), the average, scaled expression level was 2.29 for *Bmp4*, 0.83 for *Fgf8*, 1.92 for *Grem1*, 2.32 for *Shh*, and 0.02 for *Gli3*.

Statistical tests reveal that the mean-standardized variances of expression levels significantly differ among genes in the earlier (ES, E10-E11; Bartlett’s Test, F-ratio = 8.614, DF = 4, *P* < 0.001*) and later (LS, E11-E13; Bartlett’s Test, F-ratio = 5.823, DF = 4, *P* < 0.001*) stages of development. In the ES, *Shh* displays the highest average mean-standardized variance (coefficient of variation, CoV) (0.847), followed by *Bmp4* (0.567), *Grem1* (0.531), *Fgf8* (0.474), and *Gli3* (0.380). *Fgf8* displays the highest average CoV in the LS (1.701), followed by *Shh* (1.523), *Bmp4* (1.037), *Gli3* (0.910), and *Grem1* (0.703).

Litter membership also has the power to significantly explain the variance in expression levels in a given gene (e.g., *Bmp4*) that are observed among individuals (ANOVA; *Bmp4* F-ratio = 2.379, DF = 8, *P* = 0.026*; *Gli3*, F-ratio = 5.742, DF = 8, *P* = < 0.001*; *Grem1*, F-ratio = 4.412, DF = 8, *P* = < 0.001*; *Fgf8*, F-ratio = 7.097, DF = 8, *P* < 0.001*; *Shh* F-ratio = 2.162, DF = 8, *P* = 0.043*).

### Relationship between model predictions and gene expression variation among individuals

We next compared the among-individual, standardized variation in the expression level of a gene *in vivo* (CoV, from qPCR) with the: (1) number of genes whose removal alters expression of the gene in question (i.e., alters expression level by 10% or more), and (2) number of genes that exhibit expression changes when the gene in question is removed (from the simulation analyses).

**Fig 3:**
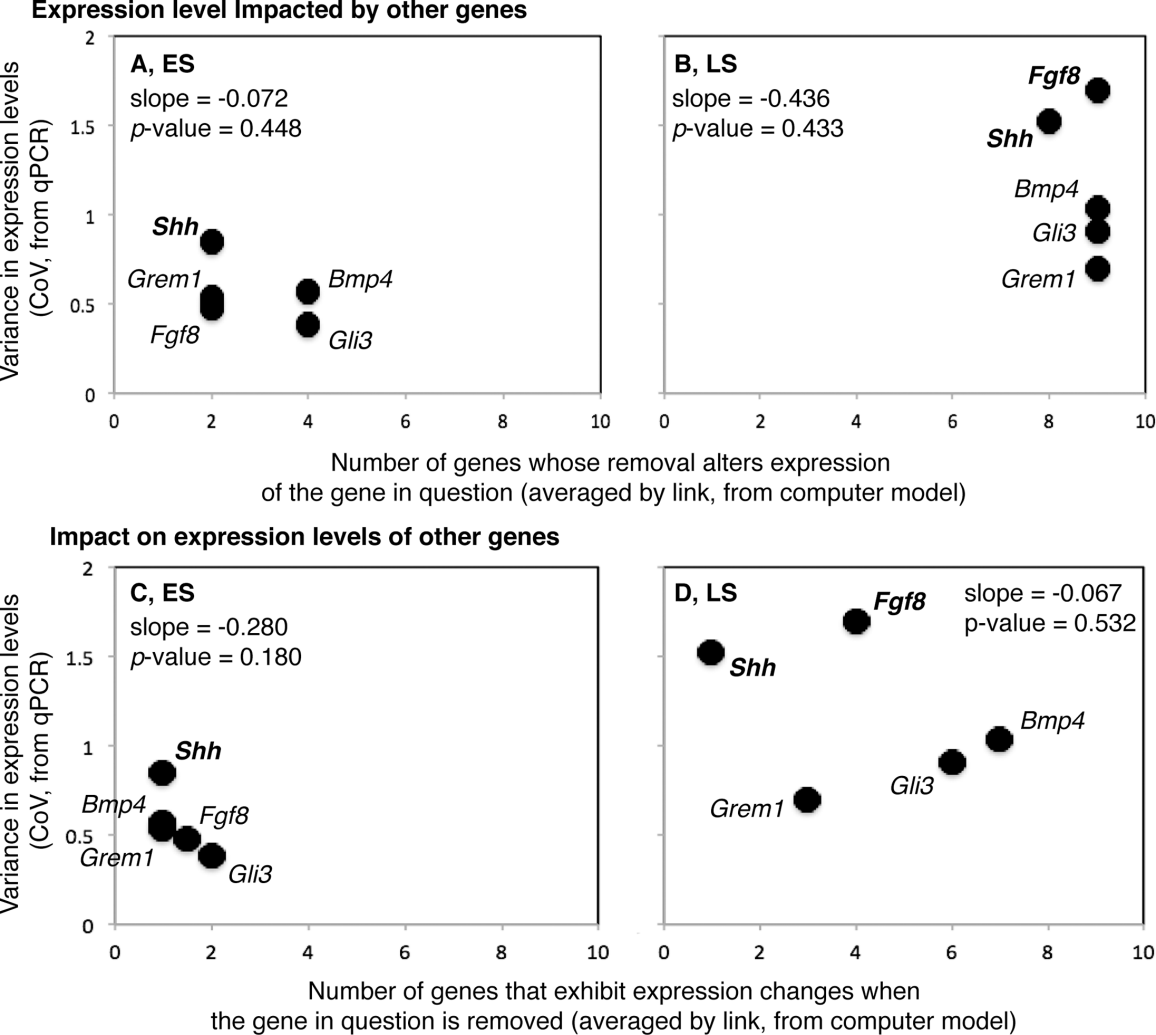
Relationships between among individual variation in gene expression (Y-axes) and gene sensitivity to network perturbation (X-axes; A and B) and ability to impact the network when perturbed (X-axes; C and D) are shown for the early (ES; A and C) and late (LS; B and D) stages of limb development. The scale for the Y-axis is the same for all plots, and the scale along the X-axis is the same for A and B, and for C and D. Variance in gene expression level tends to be lower for ES than LS genes. Variance in gene expression level is also more strongly correlated with gene sensitivity in ES genes, and gene impact in LS genes. Overall, the relationship between variance in gene expression and gene sensitivity tends to be more positive, and the relationship between variance in gene expression and gene impact more negative.

For both the ES and the LS, neither the relationships between the number of genes whose removal alters expression of the gene in question (#1) and the CoV (ES—Least-Squares Regression, R^2^ = 0.202, *P* = 0.448; LS—R^2^ = 0.214, *P* = 0.433), nor the relationships between the number of genes that exhibit expression changes when the gene in question is removed (#2) and CoV (ES—R^2^ = 0.503, *P* = 0.180; LS—R^2^ = 0.142, *P* = 0.532) are significant (Fig 3).

For both the ES and LS, *Shh* is among the genes that are the least sensitive to perturbations in other genes, has a relatively low impact on the expression of other genes when altered, and displays a relatively high CoV. The opposite is true for *Gli3R* during the ES. *Fgf8* displays the highest CoV during the LS and is highly sensitive to perturbations in other genes, like all LS genes.

Given the large difference in the percentage of possible interactions that are affected by computational alterations in the ESN and LSN (18% and 62%, respectively), we next compared the level of variation in measured gene expression during early (ES) and later (LS) development (from qPCR). For every model gene (5 of 5), the average CoV is greater later than earlier in development (*P* = 0.031*) (Fig 3). When the variation around the averages is taken into account using a resampling technique, the average CoV remains significantly greater later than earlier in development for four of the five genes (*Bmp4*, *P* = 0.028*; *Gli3*, *P* < 0.001*; *Grem1*, *P* = 0.024*; *Fgf8*, *P* < 0.001*). Only for *Shh*, a gene with among the highest CoV in both the early and later stages, does the average CoV does not remain significantly greater later than earlier in development (*P* = 0.092). The average CoV of the housekeeping gene *β-actin* does not significantly differ during earlier (0.832) and later (0.929) development (*P* < 0.217).

### Gene expression variation among species (RNASeq)

**Fig 4:**
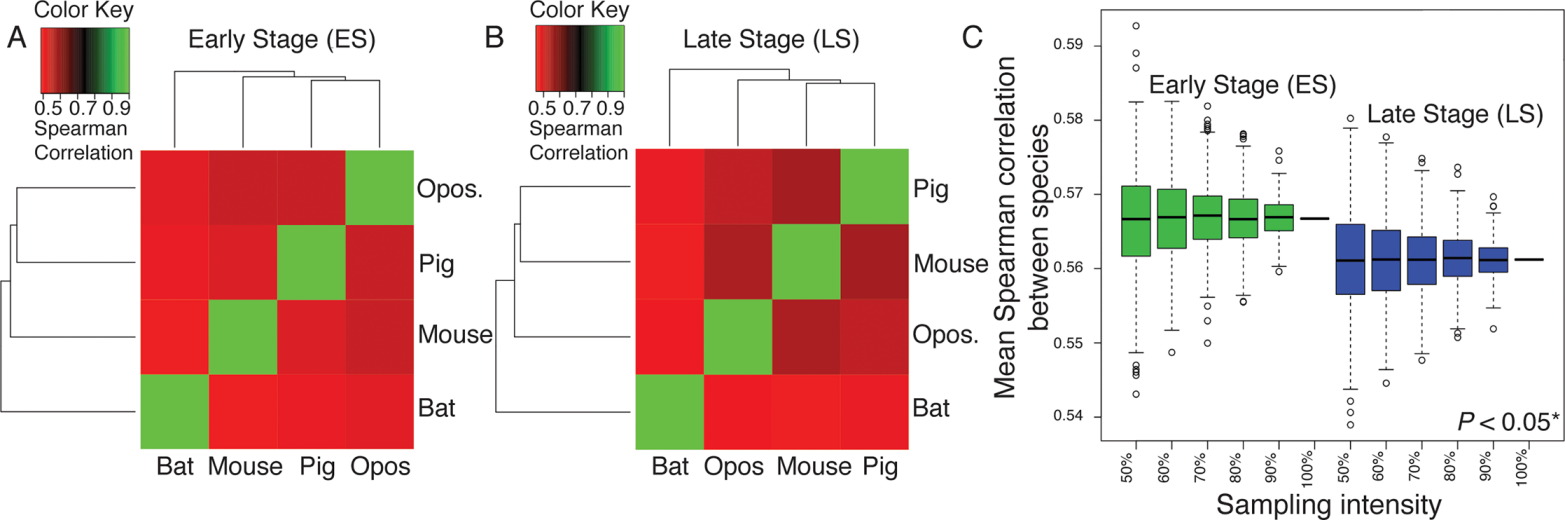
Overall patterns of gene expression are positively correlated among all examined mammals during the Early (ES; A) and Late (LS; B) stages of limb development. Opos. = opossum. (C) The Mean Spearman correlation of gene expression patterns between species is higher for the Early (ES) than Late (LS) stage of limb development. This difference is significant (*P* < 0.05) when more than 90% of orthologous genes are sampled (indicated by asterisks). Whiskers represent the 95% confidence intervals for the data.

To calculate gene expression variation among species, we first generated transcriptomic libraries for bat (*Carollia perspicillata*), opossum (*Monodelphis domestica*), pig (*Sus scrofa*) and mouse (*Mus musculus*) forelimbs for early (ES; early limb bud) and late (LS; paddle) limb stages. We then used a set of 6,583 genes orthologous to all four species (see S1 Methods) to calculate the among-species conservation of gene expression at each developmental stage, using the mean of all species pairwise Spearman coefficients. All resulting pairwise Spearman coefficients are positive and > 0.50, suggesting that the orthologous genes might perform similar functions between species (Fig 4A and 4B). However, the degree of gene expression conservation decreases from 0.5667 at ES to 0.5612 at LS of forelimb development. To test the robustness of this difference with respect to the selection of orthologous genes, we randomly sub-sampled 500 sets of orthologous genes at early and late stages at intensities ranging from 50 to 100% of all orthologous genes (Fig 4C). For each intensity, the distributions of gene expression conservation levels between early and late stages were significantly different (T-test, *P*-value < 0.05*). These results suggest that gene expression patterns vary more among species during the LS than ES of limb development, consistent with patterns of variation among individuals.

We also calculated the among-species conservation of mean-standardized expression at each developmental stage for the five genes that appear in both the ESN and LSN models (*Bmp4*, *Gli3*, *Grem1*, *Fgf8*, and *Shh*). At the ES, *Gli3* (standard deviation [SD] = 0.177) falls within the top 25% of conserved genes while *Shh* (SD = 0.975) and *Fgf8* (SD = 0.475) fall within the top 25% of divergent genes. *Bmp4* (SD = 0.257) and *Grem1* (SD = 0.260) fall near the middle of the range of genes. *Fgf8* (SD = 0.971) and *Shh* (SD = 1.196) are also among within the top 25% of divergent genes at the LS. However, *Grem1* (SD = 0.123) is among the 25% most conserved genes at this stage, and *Bmp4* (SD = 0.253) and *Gli3* (SD = 0.235) fall near the middle of the range of genes.

**Fig 5:**
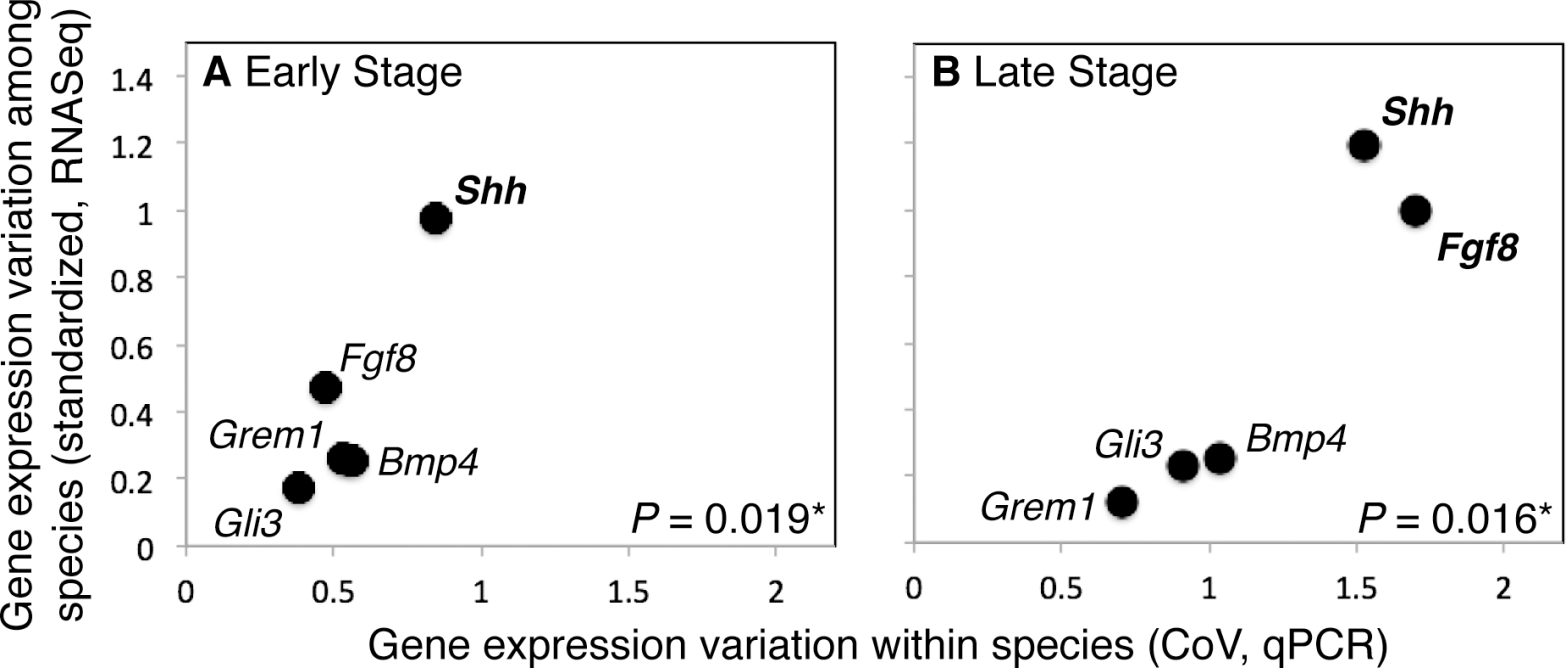
Variation in gene expression levels within a population of mice and among mammalian species are positively correlated during the Early (ES; A) and Late (LS; B) stages of limb development. *Shh* also displays the most variation in gene expression levels within and among species in both the ES and LS.

Average divergence level and average variation in expression levels among mouse individuals (CoV, as measured with qPCR) are positively correlated for the ES (R = 0.906) and LS (R = 0.854) (Fig 5), and the correlation between divergence level and CoV is significant for the ES (*P* = 0.019*) and LS (*P* = 0.016*) after bootstrapping.

## Discussion

The results reported here suggest that the structure of the early stage network (ESN) renders it more robust to perturbation than the later stage network (LSN). Findings also suggest that among individual variation in expression levels is lower for genes regulating early (ES) than late (LS) limb development, and that gene expression levels are heritable. Results of this study also suggest that the expression levels of genes at early stages generally vary less among species. Additionally, results suggest that among individual and among species variation in the expression levels of several model genes are significantly correlated for the early and later stages of limb development. Taken together, these findings suggest a scenario in which a robust ESN buffers among individual variation in gene expression early in limb development, and, as variation is a prerequisite of evolution by natural selection, limits the evolution of early limb development among species. The findings of this study are therefore consistent with the hypotheses that: (1) the structure of the early limb gene network influences the distribution of variation in mammalian limbs among individuals and over evolutionary time [21–23,38–43], and, more generally, (2) the process of early limb development generally precludes the random accumulation of variation in gene expression across the network [21,44]. Results of this study are also consistent with a scenario in which species-specific differences accumulate as development progresses. However, it is important to note that the results of this study are based on a limited number of RNASeq samples per species. Analyses of additional samples are needed to determine the degree to which the RNASeq-driven results of this study are robust to experimental variation among samples. Furthermore, while the network models used in this study are based on solid experimental data [9, 13], the results of this study are only as accurate as the network models being used.

This study did not find a significant correlation between individual gene’s sensitivity to network perturbation or impact on the network when perturbed and variation in expression among individuals. This result could stem from the lack of a relationship between these variables, or from the incompleteness of the limb gene network used in this study. However, this study did find evidence that variation in expression level significantly differs among genes, with some genes being more variable among individuals in a population, and others less so. *Shh* displays among the greatest variation in expression levels during both the early and later stages of limb development among individuals and species, and has among the least impact on the system when altered. During the early stages of limb development, *Gli3* displays the least variation in expression levels and has among the greatest effect on the system when altered.

As population-level variation provides a necessary prerequisite for evolution by natural selection, we might expect genes with the greatest expression variation among individuals to also display the most variation in expression among species. In this study this would be late stage *Shh*, *Bmp4*, and *Fgf8*. This study did find a significant correlation between the variation in the expression of these and the other key model genes among individuals and species during late limb development. Furthermore, of the few studies that have compared gene expression among mammalian limbs, a disproportionally high number have found differences in later stage *Shh*, *Bmp4*, and *Fgf8* expression among species. Evolutionary changes in *Bmp4* expression contribute to digit reduction in horses and jerboa, while evolutionary changes in *Shh* signaling contribute to digit reduction in pigs [45]. A broader initial range and secondary redeployment of *Shh* signaling helps generate the unique phenotype of the bat wing [46], and hind limb loss in dolphins is initiated by a disruption in *Shh* signaling [47]. *Shh* signaling is also activated exceptionally early during the rapid outgrowth of opossum forelimbs [48,49]. *Fgf8* expression is higher in the AER of bat wings than mouse limbs and is also secondarily redeployed later in bat wing development [50]. However, genes beyond *Shh*, *Bmp4*, and *Fgf8* also display expression differences in mammalian limbs. For example, the expression of 5’ *Hox A/D* genes differs in bat and kangaroo limbs [51,52], compared to mouse [53]. Clearly more studies of limb development in diverse mammalian species are needed to resolve this issue, but results to date are consistent with alterations in the expression of genes acting during late limb development (E11 to E13 in mouse) including *Shh*, *Bmp4*, and *Fgf8* frequently contributing to mammalian limb evolution.

In line with the proposed predominance of changes in late limb development in limb evolution, this study found no evidence for the existence of significant variation in the expression of early limb genes that is masked by systems-level processes. However, the expression-based findings of this study do not rule out the existence of cryptic genetic variation in genes with roles in early limb development. Cryptic genetic variation can provide a source of evolutionary potential when uncovered by environmental or genetic perturbations [54,55], and thereby can expedite evolutionary change. Thus, if the genes regulating early limb development possess significant cryptic genetic variation that has been uncovered over evolutionary time, we would expect early limb development to vary among species. Early limb development, as defined in this paper, encompasses establishment of the limb field and the initial outgrowth of the limb. The primary limb segments (e.g., stylopod, zeugopod and autopod) are also likely specified during this time [19,20]. Initial limb outgrowth appears to be generally conserved in tetrapods across large phylogenetic distances [56,57]. Initial limb outgrowth is even conserved in some tetrapods that do not possess limbs in their adult form (e.g., boas, dolphins) [47,58]. The primary segments of the limb are also broadly conserved across limbed tetrapods [8,59]. These observations together with the findings of this study suggest that the genes regulating early limb development either do not possess significant cryptic genetic variation, or that the robustness of the ESN has inhibited the ability of environmental or genetic perturbations to uncover this variation. Whichever the case, the early development of the limb appears to have been relatively conserved over the evolutionary history of tetrapods.

## Materials and Methods

### Ethics statement

All animal work was conducted according to relevant national and international guidelines. Animals were euthanized using CO2 inhalation followed by cervical dislocation. The University of Illinois IACUC approved this research (protocols #13128, 14159, 14199, 14209).

### Model construction

The starting point for the mathematical models used in this work was the seminal paper by Bénazet et al. 2009 [13]. These models were designed to pertain to the entire limb. Two interconnected feedback loops were incorporated into their model: a fast loop between *Grem1* and *Bmp4* and a slower loop between *Shh*, *Grem1* and the AER-*Fgf*’s. Following the findings reported in Sheth et al. 2013 [9], this network was divided into an Early (ESN) and Late (LSN) Stage Network and augmented to also include *Hox* genes (specifically, *Hox* A and D genes), *Gli3R*, *Fgf10*, and an as yet unidentified repressor, Repressor *X*. It is important to note that the specific genes, gene interactions, and equations that we include in our models match those presented in Bénazet et al. 2009 and Sheth et al. 2013, which are well supported by experimental, biological evidence.

Mathematical models to describe gene interactions were constructed in MATLAB and were based on ordinary differential equations, which are outlined in S1 Methods, along with the model parameters.

### Model simulations

We ran a series of simulations in MATLAB in which we removed interactions between genes. Only one interaction (i.e. link between two genes) was removed in each simulation, and removal simulations were performed for all interactions.

### Sensitivity analyses

We ran a series of simulations in MATLAB in which we varied the parameter values used in the models. Only one parameter was modified in each analysis.

### Among individual variation in gene expression

We performed a series of real-time quantitative PCR (qPCR) assays to quantify the expression levels of the genes that appear in both the ESN and LSN, namely *Bmp4*, *Gli3*, *Grem1*, *Shh*, and the AER-*Fgf Fgf8*, in the forelimbs of 71 mouse embryos (outbred ICR strain, Taconic) from 9 litters.

These litters ranged in age from E10 to E13. The limbs of the animals in these litters were staged according to Wanek’s staging guide, which divides mouse limb development into 15 stages [60]. Embryonic limbs from limb ridge (Wanek stage 1) through bud formation (Wanek stage 4; 4 stages total; E10 –E11) were grouped into an “early stage” (ES) for analyses, while limbs in the paddle stages of development (Wanek stages 5–8; 4 stages total; >E11 –E13) were grouped into a “late stage” (LS). Limb samples were evenly spread over all stages. The Coefficient of Variation (CoV), which is the standard deviation divided by the mean, was used to quantify variation in expression level for a given gene for early and later limb development [61]. Additional details for the qPCR analyses are in S1 Methods, and standard and dissociation curves for each gene are in S2 Methods.

Bartlett’s Test was used to compare the variance of expression levels in the ES and LS [61]. ANOVA was used to examine the contribution of litter membership (i.e., heredity) to observed patterns of gene expression [61].

### Relationship between model predictions and gene expression variation among individuals

The relationship between among individual variation in gene expression level with the number of genes whose removal alters expression of the gene in question by 10% or more from the default value (from simulation analyses) and whose values deviate by 10% or more from the default values when the gene in question is removed (from simulation analyses) was statistically assessed using Least-Squares Regression [61]. The average levels of variation in measured gene expression during early and later development (CoV) were statistically compared [61]. To determine the significance of the observed differences in CoV, we used a Monte Carlo approach, in which we shuffled the observed CoV from early and later development, to generate a null distribution of CoV differences between them. Specifically, we pooled all replicate CoV irrespective of developmental stage, then randomly drew, with replacement, two samples equal in size to the measured early and later samples, respectively. We used as a measure of significance the proportion of 10,000 replicates in which the difference between CoV’s of randomly shuffled samples was greater than or equal to the observed difference.

### Gene expression variation among species (RNASeq)

Embryonic mice, opossums, bats and pigs with early (ES) or late (LS) stage forelimbs were obtained from a variety of sources (see S1 Methods). Forelimbs for the ES were harvested at Stage 14 for bat, E11 for mouse, Stage 28 for opossum and E22 for pig [62–65]. For the LS, forelimbs were harvested at Stage 15 for bat, E12 for mouse, Stage 29 for opossum and E26 for pig.

Limbs were removed from embryos and stored in RNALater in -20°C until further processing. RNA was extracted from tissues using E.Z.N.A. Total RNA Kit I (OMEGA bio-tek #R6834), and converted into RNASeq libraries with the Illumina TruSeq RNA Sample Preparation Kit (Illumina RS-122-2001). Libraries were sequenced on an Illumina HiSeq 2500 housed in the Roy G. Carver Biotechnology Center at the University of Illinois. Resulting reads were processed, aligned to published genomes or *de novo* assemblies, and gene expression levels assessed (see S1 Methods). All data from this study have been deposited in the Gene Expression Omnibus (GEO) with the accession number GSE71390.

We analyzed the conservation of the gene expression profiles of bat, mouse, opossum, and pig across embryonic limb development, using the mean of all species pairwise Spearman coefficients (see S1 Methods). The relationship between these average species pairwise coefficients and among individual gene expression variation was assessed using Pearson Product-Moment Correlation [61]. To account for the variation among samples we used a bootstrap approach. Specifically, for the comparisons of the average species pairwise coefficients and among individual gene expression variation, we resampled, with replacement, the CoV’s (among individuals) and mean-standardized expression levels (among species) and recalculated the Pearson product-moment correlation coefficient (R) between the resampled CoV’s and mean-standardized expression levels. We used as a measure of significance the proportion of 10,000 replicates in which the calculated R was greater than or equal to zero. We also ordered the species pairwise Spearman coefficients for each individual gene from highest (most conserved) to lowest (most divergent).

## Supporting Information

S1 Table Full results of sensitivity analyses, including all tested parameter values and their impact on gene expression levels. (PDF)

S1 Methods More complete description of the models and methods used in this study. (DOCX)

S2 Methods Standard and dissociation curves for qPCR for the genes examined in this study. (DOCX)

## References

[1] Draghi J, Parsons T, Wagner G, Plotkin J (2010) Mutational robustness can facilitate adaptation. Nature 463: 353–355. 10.1038/nature08694 20090752 PMC3071712

[2] Parsons KJ, Marquez E, Albertson RC (2012) Constraint and opportunity: the genetic basis and evolution of modularity in the cichlid mandible. Am Nat 179: 64–78. 10.1086/663200 22173461

[3] Wagner GP (1988) The influence of variation and developmental constraints on the rate of multivariate phenotypic evolution. J Evol Biol 1: 45–66.

[4] Darwin CR (1869) Origin of the species by means of natural selection, or the preservation of favoured races in the struggle for life London, England: John Murray. 282 p.

[5] Goldschmidt RB (1940) The material basis of evolution New Haven, CT: Yale University Press. 436 p.

[6] Waddington CH (1942) Canalization of development and the inheritance of acquired characteristics. Nature 150: 563–565.

[7] Pigliucci M (2009) An extended synthesis for evolutionary biology. Ann N Y Acad Sci 1168: 218–228. 10.1111/j.1749-6632.2009.04578.x 19566710

[8] Polly PD (2007) Limbs in mammalian evolution In: Hall BK, editor. Fins into Limbs: Evolution, Development, and Transformation. Chicago: University of Chicago Press pp. 245–268.

[9] Sheth R, Gregoire D, Dumouchel A, Scotti M, Pham J, et al (2013) Decoupling the function of *Hox* and *Shh* in developing limb reveals multiple inputs of *Hox* genes on limb growth. Development 140: 2130–2138. 10.1242/dev.089409 23633510

[10] Rabinowitz AH, Vokes SA (2012) Integration of the transcriptional networks regulating limb morphogenesis. Dev Biol 368: 165–180. 10.1016/j.ydbio.2012.05.035 22683377

[11] Butterfield NC, McGlinn E, Wicking C (2010) The molecular regulation of vertebrate limb patterning. Curr Top Dev Biol 90: 319–341. 10.1016/S0070-2153(10)90009-4 20691854

[12] Zeller R, Lopez-Rios J, Zuniga A (2009) Vertebrate limb bud development: moving towards integrative analysis of organogenesis. Nat Rev Genet 10: 845–858. 10.1038/nrg2681 19920852

[13] Bénazet JD, Bischofberger M, Tiecke E, Goncalves A, Martin JF, et al (2009) A self-regulatory system of interlinked signaling feedback loops controls mouse limb patterning. Science 323: 1050–1053. 10.1126/science.1168755 19229034

[14] Lewandoski M, Sun X, Martin GR (2000) *Fgf8* signalling from the AER is essential for normal limb development. Nat Genet 26: 460–463. 11101846 10.1038/82609

[15] Moon AM, Capecchi MR (2000) *Fgf8* is required for outgrowth and patterning of the limbs. Nat Genet 26: 455–459. 11101845 10.1038/82601 PMC2001274

[16] Sekine K, Ohuchi H, Fujiwara M, Yamasaki M, Yoshizawa T, et al (1999) *Fgf10* is essential for limb and lung formation. Nat Genet 21: 138–141. 9916808 10.1038/5096

[17] Sun X, Mariani FV, Martin GR (2002) Functions of *Fgf* signalling from the apical ectodermal ridge in limb development. Nature 418: 501–508. 12152071 10.1038/nature00902

[18] Pajni-Underwood S, Wilson CP, Elder C, Mishina Y, Lewandoski M (2007) *Bmp* signals control limb bud interdigital programmed cell death by regulating *Fgf* signaling. Development 134: 2359–2368. 17537800 10.1242/dev.001677

[19] Cooper KL, Hu JK, ten Berge D, Fernandez-Teran M, Ros MA, et al (2011) Initiation of proximal-distal patterning in the vertebrate limb by signals and growth. Science 332: 1083–1086. 10.1126/science.1199499 21617075 PMC3258580

[20] Barna M, Pandolfi PP, Niswander L (2005) *Gli3* and *Plzf* cooperate in proximal limb patterning at early stages of limb development. Nature 436: 277–281. 16015334 10.1038/nature03801

[21] Garfield DA, Runcie DE, Babbitt CC, Haygood R, Nielsen WJ, et al (2013) The impact of gene expression variation on the robustness and evolvability of a developmental gene regulatory network. PLoS Biol 11: 1–16. 10.1371/journal.pbio.1001696 PMC3812118 24204211

[22] Galis F, Jacques JMvA, Metz JAJ (2001) Why five fingers? Evolutionary constraints on digit numbers. Trends Ecol Evol 16: 637–646.

[23] Davidson EH, Erwin DH (2006) Gene regulatory networks and the evolution of animal body plans. Science 311: 796–800. 16469913 10.1126/science.1113832

[24] von Baer KE (1828) Entwicklungsgeschichte der Thiere: Beobachtung und Reflexion. Konigsberg: Borntrager. 264 p.

[25] Raff RA (1996) The shape of life: genes, development, and the evolution of animal form Chicago: University of Chicago Press. 544 p.

[26] Carroll SB (2005) Evolution at two levels: On genes and form. PLoS Biol 3: 1159–1166. 10.1371/journal.pbio.0030245 PMC1174822 16000021

[27] Kalinka AT, Tomancak P (2012) The evolution of early animal embryos: conservation or divergence? Trends Ecol Evol 27: 385–393. 10.1016/j.tree.2012.03.007 22520868

[28] Duboule D, Wilkins AS (1998) The evolution of 'bricolage'. Trends Genet 14: 54–59. 9520598 10.1016/s0168-9525(97)01358-9

[29] Hinman VF, Nguyen AT, Cameron RA, Davidson EH (2003) Developmental gene regulatory network architecture across 500 million years of echinoderm evolution. Proc Natl Acad Sci U S A 100: 13356–13361. 14595011 10.1073/pnas.2235868100 PMC263818

[30] Lemons D, McGinnis W (2006) Genomic evolution of *Hox* gene clusters. Science 313: 1918–1922. 17008523 10.1126/science.1132040

[31] Garstang W (1922) The theory of recapitulation: a critical restatement of the Biogenetic law. J Exp Zool 291: 195–204.

[32] de Beer GR (1954) Embryos and Ancestors, Revised edition Oxford: Oxford University Press. 136 p.

[33] Reidl R (1978) Order in living organisms: A systems analysis of evolution New York, New York: Wiley. 313p.

[34] Waddington CH (1942) Canalization of development and the inheritance of acquired characters. Nature 150: 563–565. 10.1038/1831654a0 13666847

[35] Ciliberti S, Martin OC, Wagner A (2007) Innovation and robustness in complex regulatory gene networks. Proc Natl Acad Sci U S A 104: 13591–13596. 17690244 10.1073/pnas.0705396104 PMC1959426

[36] Gursky VV, Surkova SY, Samsonova MG (2012) Mechanisms of developmental robustness. Biosystems 109: 329–335. 10.1016/j.biosystems.2012.05.013 22687821

[37] Wagner A (2011) Genotype networks shed light on evolutionary constraints. Trends Ecol Evol 26: 577–584. 10.1016/j.tree.2011.07.001 21840080

[38] He J, Deem MW (2010) Hierarchical evolution of animal body plans. Dev Biol 337: 157–161. 10.1016/j.ydbio.2009.09.038 19799894

[39] Erwin DH, Davidson EH (2009) The evolution of hierarchical gene regulatory networks. Nat Rev Genet 10: 141–148. 10.1038/nrg2499 19139764

[40] Peter IS, Davidson EH (2011) Evolution of gene regulatory networks controlling body plan development. Cell 144: 970–985. 10.1016/j.cell.2011.02.017 21414487 PMC3076009

[41] Artieri CG, Haerty W, Singh RS (2009) Ontogeny and phylogeny: molecular signatures of selection, constraint, and temporal pleiotropy in the development of *Drosophila* . BMC Biol 7: 42 10.1186/1741-7007-7-42 19622136 PMC2722573

[42] Roux J, Robinson-Rechavi M (2008) Developmental constraints on vertebrate genome evolution. PLoS Genet 4: e1000311 10.1371/journal.pgen.1000311 19096706 PMC2600815

[43] Davidson EH (2010) Emerging properties of animal gene regulatory networks. Nature 468: 911–920. 10.1038/nature09645 21164479 PMC3967874

[44] Landry CR, Castillo-Davis CI, Ogura A, Liu JS, Hartl DL (2007) Systems-level analysis and evolution of the phototransduction network in *Drosophila* . Proc Natl Acad Sci U S A 104: 3283–3288. 17360639 10.1073/pnas.0611402104 PMC1805570

[45] Cooper K, Sears KE, Uygur A, Maier J, Stephan-Backowski K, et al (2014) Patterning and post-patterning modes of evolutionary digit loss in mammals. Nature 511: 41–45. 10.1038/nature13496 24990742 PMC4228958

[46] Hockman D, Cretekos CJ, Mason MK, Behringer RR, Jacobs DS, et al (2008) A second wave of *sonic hedgehog* expression during the development of the bat limb. Proc Natl Acad Sci U S A 105: 16982–16987. 10.1073/pnas.0805308105 18957550 PMC2579364

[47] Thewissen JGM, Cohn MJ, Stevens ME, Bajpai S, Heyning J, et al (2006) Developmental basis for hind-limb loss in dolphins and origin of the cetacean bodyplan. Proc Natl Acad Sci U S A 103: 8414–8418. 16717186 10.1073/pnas.0602920103 PMC1482506

[48] Keyte AL, Smith KK (2010) Developmental origins of precocial forelimbs in marsupial neonates Development 137: 4283–4294. 10.1242/dev.049445 21098569

[49] Sears KE, Doroba CK, Xie D, Zhong S (2012) Molecular determinants of marsupial limb integration and constraint In: Müller J, Asher R, editors. From Clone to Bone: The Synergy of Morphological and Molecular Tools in Paleobiology. Cambridge: Cambridge University Press pp. 257–278.

[50] Cretekos CJ, Deng JM, Green ED, Rasweiler JJ, Behringer RR (2007) Isolation, genomic structure and developmental expression of *Fgf8* in the short-tailed fruit bat, *Carollia perspicillata* . Int J Dev Biol 51: 333–338. 17554686 10.1387/ijdb.062257cc

[51] Chen CH, Cretekos CJ, Rasweiler JJ, Behringer RR (2005) *Hoxd13* expression in the developing limbs of the short-tailed fruit bat, *Carollia perspicillata* . Evolution and Development 7: 130–141. 15733311 10.1111/j.1525-142X.2005.05015.x

[52] Ray R, Capecchi MR (2008) An examination of the chiropteran *HoxD* locus from an evolutionary perspective. Evolution and Development 10: 657–670. 10.1111/j.1525-142X.2008.00279.x 19021736

[53] Chew KY, Yu H, Pask AJ, Shaw G, Renfree MB (2012) *Hoxa13* and *Hoxd13* expression during development of the syndactylous digits in the marsupial *Macropus eugenii* . BMC Dev Biol 12: 2 10.1186/1471-213X-12-2 22235805 PMC3268106

[54] Gibson G, Dworkin I (2004) Uncovering cryptic genetic variation. Nat Rev Genet 5: 681–690. 15372091 10.1038/nrg1426

[55] Rohner N, Jarosz DF, Kowalko JE, Yoshizawa M, Jeffery WR, et al (2013) Cryptic variation in morphological evolution: *HSP90* as a capacitor for loss of eyes in cavefish. Science 342: 1372–1375. 10.1126/science.1240276 24337296 PMC4004346

[56] Zeller R (2010) The temporal dynamics of vertebrate limb development, teratogenesis and evolution. Curr Opin Genet Dev 20: 384–390. 10.1016/j.gde.2010.04.014 20537528

[57] Sears KE, Patel A, Hübler M, Cao X, VandeBerg JL, et al (2012) Disparate *Igf1* expression and growth in the fore- and hind limbs of a marsupial (*Monodelphis domestica*). Exp Zool Part B 318: 279–293. 10.1002/jez.b.22444 22821864

[58] Cohn MJ, Tickle C (1999) Developmental basis of limblessness and axial patterning in snakes. Nature 399: 474–479. 10365960 10.1038/20944

[59] Shubin N, Tabin C, Carroll S (1997) Fossils, genes and the evolution of animal limbs. Nature 388: 639–648. 9262397 10.1038/41710

[60] Wanek N, Muneoka K, Holler-Dinsmore G, Burton R, Bryant SV (1989) A staging system for mouse limb development. J Exp Zool 249: 41–49. 2926360 10.1002/jez.1402490109

[61] Sokal RR, Rohlf FJ (1995) Biometry. New York: W.H. Freeman and Company. 880 p.

[62] Cretekos CJ, Weatherbee SD, Chen CH, Badwaik NK, Niswander L, et al (2005) Embryonic staging system for the short-tailed fruit bat, *Carollia perspicillata*, a model organism for the mammalian order Chiroptera, based upon timed pregnancies in captive-bred animals. Dev Dyn 233: 721–738. 15861401 10.1002/dvdy.20400

[63] Mate KE, Robinson ES, VandeBerg JL, Pederson RA (1994) Timetable of *in vivo* embryonic development in the gray short-tailed opossum (*Monodelphis domestica*). Mol Reprod Dev 39: 365–374. 7893485 10.1002/mrd.1080390404

[64] McCrady E (1938) The embryology of the Opossum Philadelphia: Wistar Institute of Anatomy and Biology. 233 p.

[65] Butler H, Juurlink BHJ (1987) An Atlas for Staging Mammalian and Chick Embryos. Boca Raton, Florida: CRC Press. 218 p.

