# Supplementary material for: The Relationship between Gene Network Structure and Expression Variation among Individuals and Species
Source: PLoS Genet. 2015 Aug 28;11(8):e1005398. doi: 10.1371/journal.pgen.1005398 (PMC4552942; doi:10.1371/journal.pgen.1005398)
Supplement: S1 Table — (PDF) [file pgen.1005398.s001.pdf]

Supplementary Table 1. Results of the sensitivity analyses. Default parameters are shaded in grey. Altered expression levels that differ from default levels by more than 10% are indicated by asterisks.

ESN, AER-*Fgf*'s	pAF1=0.05	pAF1=0.25	pAF1=0.45	pAF2=0.05	pAF2=0.25	pAF2=0.45	aAF=0.05	aAF=0.25	aAF=0.45
AER-*Fgf*'s	1.19*	1.98	2.77*	1.95	1.98	1.98	6.56*	1.98	1.11*
Bmp4	0.01	0.01	0.01	0.01	0.01	0.01	0.01	0.01	0.01
Fgf10	1.94	1.98	1.98	1.19*	1.98	2.77*	1.98	1.98	1.94
Gli3R	0.01*	0.01	0.01	0.01	0.01	0.01	0.01	0.01	0.01
Grem1	1.99	1.99	1.99	1.99	1.99	1.99	1.99	1.99	1.99
Hox A/D	1.80	1.80	1.80	1.80	1.80	1.80	1.80	1.80	1.80
Shh	1.92	1.97	1.98	1.97	1.97	1.97	1.99	1.97	1.92

ESN, AER-*Fgf*'s	K5=0	K5=0.4	K5=0.8	K5=1.2	K6=0	K6=0.4	K6=0.8	K6=1.2
AER-*Fgf*'s	1.99	1.98	1.92	1.79	0.99*	1.98	1.98	1.99
Bmp4	0.01	0.01	0.01	0.01	0.01	0.01	0.01	0.01
Fgf10	1.98	1.98	1.97	1.97	1.92	1.98	1.98	1.98
Gli3R	0.01	0.01	0.01	0.01	0.01	0.01	0.01	0.01
Grem1	1.99	1.99	1.99	1.99	1.99	1.99	1.99	1.99
Hox A/D	1.80	1.80	1.80	1.80	1.80	1.80	1.80	1.80
Shh	1.97	1.97	1.97	1.96	1.90	1.97	1.97	1.97

LSN, AER-*Fgf*'s	pAF=0.05	pAF=0.25	pAF=0.5	pAF=0.75	aAF=0.05	aAF=0.25	aAF=0.45	aAF=0.65	aAF=0.85	K5=0	K5=0.4	K5=0.6	K5=1.2
AER-*Fgf*'s	0.15*	0.57*	0.79	0.97*	6.48*	0.79	0.43*	0.35*	0.36*	0.00*	0.72	0.79	1.15*
Bmp4	0.40*	0.72*	0.92	1.02	1.07*	0.92	0.82*	0.71*	0.56*	0.40*	0.68*	0.92	1.30*
Gli3R	1.00*	0.01*	0.01	0.01	0.01*	0.01	0.02*	0.02*	0.03*	1.00*	0.01	0.01	0.01
Grem1	0.64*	0.36	0.31	0.28	0.27	0.31	0.33	0.37*	0.44*	0.64*	0.38	0.31	0.22*
Hox A/D	0.11*	1.85	1.95	1.97	2.00	1.95	1.77	1.62*	1.58*	0.06*	1.93	1.95	1.98
Rep *X*	0.00*	0.05	0.05	0.05	0.05	0.05	0.05	0.05	0.05	0.00*	0.05	0.05	0.06
Shh	0.02*	1.53*	1.77	1.85	1.96*	1.77	1.42*	1.25*	1.19*	0.00*	1.74	1.77	1.86

LSN, AER-*Fgf*'s	K6=0	K6=0.4	K6=0.6	K6=1.2	K7=0	K7=0.4	K7=0.6	K7=0.8	K7=1.2	K8=0	K8=0.4	K8=0.8	K8=1.2
AER-*Fgf*'s	0.72	0.79	0.80	0.71*	2.00*	0.79	0.51*	0.86	0.13*	0.60*	0.79	0.76	0.74
Bmp4	0.97	0.92	0.91	0.98	0.00*	0.92	1.15*	0.89	1.45*	1.06*	0.92	0.94	0.95
Gli3R	0.00*	0.01	0.03*	0.22*	0.01*	0.01	0.01*	0.01	1.00*	0.02*	0.01	0.01	0.01
Grem1	0.30	0.31	0.31	0.29	0.19*	0.31	0.37*	0.61*	0.87*	0.27*	0.31	0.30	0.30
Hox A/D	1.93	1.95	1.95	1.80	1.99	1.95	1.85	0.96*	0.09*	0.89*	1.95	1.94	1.94
Rep *X*	0.05	0.05	0.05	0.05	0.05	0.05	0.05	0.05*	0.00*	0.05	0.05	0.05	0.05
Shh	1.73	1.77	1.77	1.71	1.93	1.77	1.56*	1.80	0.01*	1.40*	1.77	1.75	1.74

ESN, *Bmp4*	pB=0.05	pB=0.25	pB=0.45	aB=0.05	aB=0.25	aB=0.45	aB=0.65	aB=0.85	K10=0.00	K10=0.4	K10=0.8	K10=1.2
AER-*Fgf*'s	1.98	1.98	1.98	1.39*	1.98	1.98	1.99	1.99	1.98	1.98	1.97	1.91
Bmp4	0.00*	0.01	0.02*	0.39*	0.01	0.00*	0.00*	0.00*	0.00*	0.01	0.06*	0.15*
Fgf10	1.98	1.98	1.98	1.95	1.98	1.98	1.98	1.98	1.98	1.98	1.97	1.97
Gli3R	0.01	0.01	0.01	0.01	0.01	0.01	0.01	0.01	0.01	0.01	0.01	0.01
Grem1	1.99	1.99	1.99	2.75*	1.99	1.98	1.98	1.98	1.98	1.99	2.01	2.12
Hox A/D	1.80	1.80	1.80	1.80	1.80	1.80	1.80	1.80	1.80	1.80	1.80	1.80
Shh	1.97	1.97	1.97	1.94	1.97	1.97	1.97	1.97	1.97	1.97	1.97	1.97

LSN, *Bmp4*	pB=0.05	pB=0.25	pB=0.45	aB=0.05	aB=0.125	aB=0.45	aB=0.65	aB=0.85	K10=0	K10=0.1	K10=0.7	K10=1.2
AER-*Fgf*'s	1.90*	0.79	0.49*	1.83*	0.79	1.49*	1.75*	1.86*	0.05*	0.79	2.00*	2.00*
Bmp4	0.25*	0.92	1.32*	0.34*	0.92	0.46*	0.34*	0.27*	2.00*	0.92	0.02*	0.02*
Gli3R	0.01*	0.01	0.01*	0.01	0.01	0.01	0.01	0.01*	1.00*	0.01	0.01*	0.01*
Grem1	0.23*	0.31	0.35*	0.66*	0.31	0.21*	0.19*	0.18*	0.00*	0.31	1.91*	1.91*
Hox A/D	1.99	1.95	1.85	1.99	1.95	1.98	1.99	1.99	0.06*	1.95	1.99	1.99
Rep *X*	0.05	0.05	0.05	0.04*	0.05	0.06	0.06	0.06	0.00*	0.05	0.05	0.05
Shh	1.92	1.77	1.58*	1.92	1.77	1.90	1.91	1.92	0.00*	1.77	1.93	1.93

ESN, *Fgf10*	pF1=0.05	pF1=0.25	pF1=0.45	pF2=0.05	pF2=0.25	pF2=0.45	aF=0.05	aF=0.25	aF=0.45	aF=0.65	aF=0.85
AER-*Fgf*'s	1.95	1.98	1.98	1.95	1.98	1.98	1.99	1.98	1.94	1.86	1.74
Bmp4	0.01	0.01	0.01	0.01	0.01	0.01	0.01	0.01	0.01	0.01	0.01
Fgf10	1.19*	1.98	2.76*	1.19*	1.98	2.77*	7.05*	1.98	1.10*	0.08*	0.58*
Gli3R	0.01	0.01	0.01	0.01	0.01	0.01	0.01	0.01	0.01	0.01	0.01
Grem1	1.99	1.99	1.99	1.99	1.99	1.99	1.99	1.99	1.99	1.99	1.99
Hox A/D	1.80	1.80	1.80	1.80	1.80	1.80	1.80	1.80	1.80	1.80	1.80
Shh	1.97	1.97	1.97	1.97	1.97	1.97	1.97	1.97	1.97	1.97	1.97

ESN, *Fgf10*	K7=0	K7=0.4	K7=0.8	K7=1.2	K8=0.0	K8=0.4	K8=0.8	K8=1.2
AER-*Fgf*'s	1.98	1.98	1.98	1.98	1.98	1.98	1.98	1.98
Bmp4	0.01	0.01	0.01	0.01	0.01	0.01	0.01	0.01
Fgf10	1.99	1.98	1.90	1.75*	1.99	1.98	1.91	1.79
Gli3R	0.01	0.01	0.01	0.01	0.01	0.01	0.01	0.01
Grem1	1.99	1.99	1.99	1.99	1.99	1.99	1.99	1.99
Hox A/D	1.80	1.80	1.80	1.80	1.80	1.80	1.80	1.80
Shh	1.97	1.97	1.97	1.97	1.97	1.97	1.97	1.97

ESN, *Gli3R*	pR=0.05	pR=0.25	pR=0.45	aR=0.05	aR=0.25	aR=0.45	aR=0.65	aR=0.85	K9=0.00	K9=0.4	K9=0.8	K9=1.2
AER-*Fgf*'s	1.98	1.98	1.98	1.98	1.98	1.98	1.98	1.98	1.98	1.98	1.98	1.98
Bmp4	0.01	0.01	0.01	0.03*	0.01	0.01	0.01	0.01	0.01	0.01	0.01	0.01
Fgf10	1.98	1.98	1.98	1.98	1.98	1.98	1.98	1.98	1.98	1.98	1.98	1.98
Gli3R	0.00*	0.01	0.02*	0.47*	0.01	0.00*	0.00*	0.00*	0.00*	0.01	0.07*	0.20*
Grem1	1.99	1.99	1.98	1.43*	1.99	1.99	1.99	1.97	1.99	1.99	1.97	1.89
Hox A/D	1.80	1.80	1.80	1.80	1.80	1.80	1.80	1.80	1.80	1.80	1.80	1.80
Shh	1.97	1.97	1.97	1.97	1.97	1.97	1.97	1.97	1.97	1.97	1.97	1.97

LSN, *Gli3R*	pR=0.05	pR=0.25	pR=0.45	aR=0.05	aR=0.25	aR=0.45	aR=0.65	aR=0.85	K9=0	K9=0.4	K9=0.8	K9=1.2
AER-*Fgf*'s	0.73	0.79	0.82	0.68*	0.79	0.74	0.71*	0.69*	0.72	0.79	0.78	0.77
Bmp4	0.96	0.92	0.90	0.96	0.92	0.95	0.97	0.98	0.96	0.92	0.93	0.94
Gli3R	0.00*	0.01	0.02*	0.15*	0.01	0.01*	0.00*	0.00*	0.01	0.01	0.01	0.01
Grem1	0.30	0.31	0.31	0.30	0.31	0.30	0.29	0.29	0.30	0.31	0.30	0.30
Hox A/D	1.99	1.95	1.95	1.83	1.95	1.94	1.93	1.93	2.00	1.95	1.70*	1.43*
Rep *X*	1.74	1.77	1.78	1.68	1.77	1.74	1.72	1.71	1.73	1.77	1.75	1.72
Shh	0.05	0.05	0.05	0.05	0.05	0.05	0.05	0.05	0.05	0.05	0.05	0.05

ESN, *Grem1*	pG1=0.05	pG1=0.25	pG1=0.45	pG2=0.05	pG2=0.25	pG2=0.45	pG3=0.05	pG3=0.25	pG3=0.45	aG=0.05	aG=0.25	aG=0.45	aG=0.65
AER-*Fgf*'s	1.98	1.98	1.98	1.98	1.98	1.98	1.98	1.98	1.98	1.98	1.98	1.98	1.95
Bmp4	0.04*	0.01	0.01*	0.04*	0.01	0.01*	0.01*	0.01	0.01	0.00*	0.01	0.05*	0.12*
Fgf10	1.98	1.98	1.98	1.98	1.98	1.98	1.98	1.98	1.98	1.98	1.98	1.98	1.97
Gli3R	0.01	0.01	0.01	0.01	0.01	0.01	0.01	0.01	0.01	0.01	0.01	0.01	0.01
Grem1	1.21*	1.99	2.78*	1.20*	1.99	2.78*	1.98	1.99	1.99	7.08*	1.99	1.11*	0.79*
Hox A/D	1.80	1.80	1.80	1.80	1.80	1.80	1.80	1.80	1.80	1.80	1.80	1.80	1.80
Shh	1.97	1.97	1.97	1.97	1.97	1.97	1.97	1.97	1.97	1.97	1.97	1.97	1.97

ESN, *Grem1*	aG=0.85	K0=0	K0=0.4	K0=0.8	K0=1.2	K1=0	K1=0.5	K1=0.8	K1=1.2	K2=0	K2=0.3	K2=0.8	K2=1.2
AER-*Fgf*'s	1.87	1.98	1.98	1.98	1.98	1.98	1.98	1.98	1.98	1.98	1.98	1.98	1.98
Bmp4	0.19*	0.01	0.01	0.01	0.02	0.06*	0.01	0.01	0.01	0.01*	0.01	0.01	0.01*
Fgf10	1.97	1.98	1.98	1.98	1.98	1.98	1.98	1.98	1.98	1.98	1.98	1.98	1.98
Gli3R	0.01	0.01	0.01	0.01	0.01	0.01	0.01	0.01	0.01	0.01	0.01	0.01	0.01
Grem1	0.65*	2.00	1.99	1.92	1.77*	1.01*	1.99	1.99	1.99	2.97*	1.99	1.98	1.98
Hox A/D	1.80	1.80	1.80	1.80	1.80	1.80	1.80	1.80	1.80	1.80	1.80	1.80	1.80
Shh	1.97	1.97	1.97	1.97	1.97	1.97	1.97	1.97	1.97	1.97	1.97	1.97	1.97

LSN, *Grem1*	pG1=0.05	pG1=0.20	pG1=0.45	pG2=0.05	pG2=0.30	pG2=0.45	pG3=0.05	pG3=0.20	pG3=0.45	aG=0.05	aG=0.25	aG=0.45	aG=0.65
AER-*Fgf*'s	0.51*	0.79	1.29*	0.29*	0.79	1.17*	0.11*	0.79	1.08*	0.94*	0.79	0.95*	0.19*
Bmp4	1.13*	0.92	0.65*	1.39*	0.92	0.71*	1.53*	0.92	0.72*	0.85	0.92	0.80*	1.27*
Gli3R	0.01*	0.01	0.01	0.02*	0.01	0.01	1.00*	0.01	0.01	0.01	0.01	0.01	1.00*
Grem1	0.26*	0.31	0.39*	0.21*	0.31	0.37*	0.27*	0.31	0.38*	0.32	0.31	0.35*	0.33
Hox A/D	1.85	1.95	1.98	1.55*	1.95	1.98	0.08*	1.95	1.97	1.97	1.95	1.96	0.16*
Rep *X*	0.05	0.05	0.05	0.05	0.05	0.05	0.00*	0.05	0.05	0.05	0.05	0.05	0.00*
Shh	1.55*	1.77	1.89	1.23*	1.77	1.87	0.01*	1.77	1.85	1.83	1.77	1.82	0.04*

LSN, *Grem1*	aG=0.85	K0=0	K0=0.4	K0=1.0	K0=1.2	K1=0	K1=0.4	K1=0.8	K1=1.2	K2=0	K2=0.3	K2=0.8	K2=1.2
AER-*Fgf*'s	0.10*	0.73	0.75	0.79	0.78	0.22*	0.79	0.70*	0.67*	0.99*	0.79	0.59*	0.84
Bmp4	1.59*	0.96	0.95	0.92	0.93	1.48*	0.92	0.98	1.00	0.85	0.92	1.05*	0.92
Gli3R	1.00*	0.01	0.01	0.01	0.01	0.03*	0.01	0.01	0.01	0.01	0.01	0.01	0.01
Grem1	0.26*	0.30	0.30	0.31	0.30	0.21*	0.31	0.29	0.29	0.31	0.31	0.29	0.31
Hox A/D	0.08*	1.93	1.94	1.95	1.94	1.40*	1.95	1.93	1.92	1.97	1.95	1.89	1.96
Rep *X*	0.00*	0.05	0.05	0.05	0.05	0.05	0.05	0.05	0.05	0.05	0.05	0.05	0.05
Shh	0.01*	1.74	1.75	1.77	1.76	1.11*	1.77	1.72	1.69	1.85	1.77	1.64	1.80

ESN, *Hox A/D*	l=0.05	l=0.15	l=0.30	l=0.45	l=0.60	aH=0.05	aH=0.25	aH=0.45	aH=0.65	aH=0.85
AER-*Fgf*'s	1.95	1.98	1.98	1.98	1.98	1.98	1.98	1.98	1.98	1.97
Bmp4	0.04*	0.01*	0.01	0.01	0.01	0.01	0.01	0.01	0.01*	0.02*
Fgf10	1.12*	1.76*	1.95	1.98	1.98	1.99	1.98	1.93	1.83	1.69*
Gli3R	0.04*	0.01*	0.01	0.01	0.01	0.01	0.01	0.01	0.01	0.02
Grem1	1.13*	1.78*	1.96	1.99	1.99	2.00	1.99	1.94	1.84	1.70*
Hox A/D	0.20*	0.60*	1.20*	1.80	2.40*	6.59*	1.80	1.00*	0.69*	0.53*
Shh	1.16*	1.82	1.96	1.97	1.98	1.98	1.97	1.94	1.87	1.76*

LSN, *Hox A/D*	l=0	l=0.3	l=0.6	l=0.9	l=1.2	aH=0.05	aH=0.25	aH=0.45	aH=0.65	aH=0.85
AER-*Fgf*'s	0.79	0.76	0.75	0.74	0.74	0.79	0.79	0.66*	0.55*	0.49*
Bmp4	0.92	0.94	0.95	0.95	0.95	0.92	0.92	1.01	1.10*	1.16*
Gli3R	0.01	0.01	0.01	0.01	0.01	0.01	0.01	0.01*	0.03*	0.05*
Grem1	0.31	0.30	0.30	0.30	0.30	0.31	0.31	0.28	0.26*	0.25*
Hox A/D	1.95	3.14*	4.34*	5.54*	6.74*	9.66*	1.95	1.05*	0.69*	0.51*
Rep *X*	0.05	0.05	0.05	0.05	0.05	0.05	0.05	0.05	0.05	0.05
Shh	1.77	1.78	1.78	1.78	1.78	2.81*	1.77	1.52*	1.17*	0.90*

LSN, Rep *X*	pX=0.0005	pX=0.001	pX=0.25	pX=0.45	aX=0.005	aX=0.01	aX=0.125	aX=0.45	aX=0.65	aX=0.85
AER-*Fgf*'s	2.00*	0.79	0.05*	0.05*	0.19*	0.79	2.00*	2.00*	2.00*	2.00*
Bmp4	0.02*	0.92	2.00*	2.00*	1.61*	0.92	0.02*	0.02*	0.02*	0.02*
Gli3R	0.01*	0.01	1.00*	1.00*	0.03*	0.01	0.01*	0.01*	0.01*	0.01*
Grem1	1.73*	0.31	0.00*	0.00*	0.16*	0.31	1.91*	1.91*	1.91*	1.91*
Hox A/D	1.99	1.95	0.06*	0.06*	1.34*	1.95	1.99	1.99	1.99	1.99
Rep *X*	0.03*	0.05	0.32*	0.38*	0.06*	0.05	0.01*	0.00*	0.00*	0.00*
Shh	1.93	1.77	0.00*	0.00*	1.08*	1.77	1.93	1.93	1.93	1.93

LSN, Rep *X*	K11=0	K11=0.3	K11=0.6	K11=0.8	K11=1.2
AER-*Fgf*'s	0.23*	0.79	1.14*	1.37*	1.85*
Bmp4	1.47*	0.92	0.72*	0.60*	0.34*
Gli3R	0.03*	0.01	0.01	0.01	0.01*
Grem1	0.19*	0.31	0.37*	0.41*	0.55*
Hox A/D	1.43*	1.95	1.98	1.98	1.99
Rep *X*	0.06	0.05	0.05	0.05	0.05*
Shh	1.13*	1.77	1.87	1.90	1.92

ESN, *Shh*	pS1=0.05	pS1=0.25	pS1=0.45	pS2=0.05	pS2=0.25	pS2=0.45	aS=0.05	aS=0.25	aS=0.45	aS=0.65	aS=0.85
AER-*Fgf*'s	1.98	1.98	1.98	1.98	1.98	1.98	1.98	1.98	1.98	1.98	1.98
Bmp4	0.01	0.01	0.01	0.01	0.01	0.01	0.01	0.01	0.01	0.01	0.01*
Fgf10	1.98	1.98	1.98	1.98	1.98	1.98	1.98	1.98	1.98	1.98	1.98
Gli3R	0.04*	0.01	0.01*	0.05*	0.01	0.01*	0.00*	0.01	0.05*	0.13*	0.25*
Grem1	1.98	1.99	1.99	1.98	1.99	1.99	1.99	1.99	1.98	1.96	1.86
Hox A/D	1.80	1.80	1.80	1.80	1.80	1.80	1.80	1.80	1.80	1.80	1.80
Shh	1.19*	1.97	2.76*	1.18*	1.97	2.76*	6.74*	1.97	1.10*	0.76*	0.58*

ESN, *Shh*	K3=0	K3=0.45	K3=0.8	K3=1.2	K4=0	K4=0.35	K4=0.8	K4=1.2

AER-*Fgf*'s	1.98	1.98	1.98	1.98	1.98	1.98	1.98	1.98
Bmp4	0.01	0.01	0.01	0.01	0.01	0.01	0.01	0.01
Fgf10	1.98	1.98	1.98	1.98	1.98	1.98	1.98	1.98
Gli3R	0.01	0.01	0.01	0.02	0.01	0.01	0.01*	0.02*
Grem1	1.99	1.99	1.99	1.99	1.99	1.99	1.99	1.99
Hox A/D	1.80	1.80	1.80	1.80	1.80	1.80	1.80	1.80
Shh	1.99	1.97	1.92	1.79	1.98	1.97	1.90	1.75*

LSN, *Shh*	pS1=0.05	pS1=0.25	pS1=0.45	pS2=0.05	pS2=0.25	pS2=0.45	aS=0.05	aS=0.25	aS=0.45	aS=0.65	aS=0.85
AER-*Fgf*'s	0.79	0.79	0.79	0.80	0.79	0.77	0.77	0.79	0.80	0.76	0.59*
Bmp4	0.92	0.92	0.92	0.91	0.92	0.94	0.93	0.92	0.92	0.94	1.08
Gli3R	0.04*	0.01	0.00*	0.05*	0.01	0.00*	0.00*	0.01	0.06*	0.16*	0.35*
Grem1	0.31	0.31	0.31	0.31	0.31	0.30	0.30	0.31	0.31	0.30	0.25*
Hox A/D	1.94	1.95	1.95	1.95	1.95	1.94	1.94	1.95	1.94	1.89	1.59
Rep *X*	0.05	0.05	0.05	0.05	0.05	0.05	0.05	0.05	0.05	0.05	0.05
Shh	1.12*	1.77	2.42*	1.01*	1.77	2.52*	9.09*	1.77	0.96*	0.64*	0.43*

LSN, *Shh*	K3=0	K3=0.4	K3=0.65	K3=1.2	K4=0	K4=0.4	K4=0.7	K4=1.2
AER-*Fgf*'s	0.74	0.79	0.79	0.79	0.76	0.77	0.79	0.80
Bmp4	0.95	0.92	0.92	0.92	0.94	0.94	0.92	0.91
Gli3R	0.01*	0.01*	0.01	0.02*	0.01	0.01	0.01	0.01*
Grem1	0.30	0.31	0.31	0.31	0.30	0.30	0.31	0.31
Hox A/D	1.94	1.95	1.95	1.95	1.94	1.94	1.95	1.95
Rep *X*	0.05	0.05	0.05	0.05	0.05	0.05	0.05	0.05
Shh	1.96*	1.90	1.77	1.40*	1.79	1.79	1.77	1.63
