# Supplementary material for: The Relationship between Gene Network Structure and Expression Variation among Individuals and Species
Source: PLoS Genet. 2015 Aug 28;11(8):e1005398. doi: 10.1371/journal.pgen.1005398 (PMC4552942; doi:10.1371/journal.pgen.1005398)
Supplement: S1 Methods — (DOCX) [file pgen.1005398.s002.docx]

**Supplementary Methods**

***Early Stage Model description***

Our model of the ESN (and LSN) is based on published experimental studies of genetic interactions, and as such incorporates the same genes that are included in these studies (Benazet et al., 2009 and Sheth et al, 2013). In the ESN, early expressed *Hox* genes activate *Fgf10*, *Shh* and *Grem1*. *Fgf10*, in turn, activate the AER-*Fgf*’s, which feedback to activate *Fgf10* and *Shh*. *Gli3R* is inhibited by *Shh* and inhibits *Grem1*, which inhibits *Bmp4*. *Bmp4*, however, activates *Grem1* and inhibits AER *Fgf*’s (Fig. 1A).

In our mathematical model of these interactions, *Hox* *A/D* (H) are fed into the system at a constant rate l and degrade with first order kinetics (linear degradation at a rate a_H_). The activity of *Grem1* (G) is positively controlled by the *Hox* genes and *Bmp4* (B). These interactions are modeled by Hill functions with maximal velocities p_G1_ and p_G3_ and half maximal induction concentrations K_0_ and K_2_. The half-life of *Grem1* is denoted by 1/a_G_. Also, *Grem1* is negatively regulated by *Gli3R* (R). This is again modeled by a Hill function with maximal velocity p_G2_ and half maximal induction concentration K_1_. The activity and concentration of *Shh* (S) is positively controlled by both the Aer-*Fgf*’s (AF) and *Hox A/D*. These interactions are modeled by Hill functions with maximal velocity p_S1_, p_S2_ and half maximal induction concentrations K_3,_ K_4_, respectively. The half-life of SHH is 1/a_S_. The activity and concentration of the AER-*Fgf*’s (AF) is positively controlled by *Fgf10* (F) and negatively controlled by *Bmp4*. These interactions are modeled by Hill functions with maximal velocity p_AF1_, p_AF2_ and half maximal induction concentrations K_5,_ K_6_, respectively. The half-life of the AER-*Fgf*’s is 1/a_AF_. The activity and concentration of *Fgf10* (F) is positively controlled by both *Hox A/D* and the AER-*Fgf*’s (AF). These interactions are modeled by Hill functions with maximal velocity p_F1_, p_F2_ and half maximal induction concentrations K_7,_ K_8_, respectively. The half-life of *Fgf10* is 1/a_F_. *Gli3R* (R) is inhibited by *Shh* (S) following Hill function kinetics with maximal velocity p_R_ and half maximal induction concentrations K_9_. Its half-life is equal to 1/a_R_. Finally, *Bmp4* (B) is inhibited by *Grem1* (G) following Hill function kinetics with maximal velocity p_B_ and half maximal induction concentrations K_10_. Its half-life is equal to 1/a_B_. A common Hill exponent *n* is used in all equations. The system of ordinary differential equations that describes the interactions among these genes is the following. Parameter values are given in Supplementary Table 2.

$$\begin{matrix} \frac{dH}{dt}=l-a_{H}H \\ \frac{dG}{dt}=p_{G1}\cdot\frac{H^{n}}{H^{n}+K_{0}}+p_{G2}\cdot\frac{K_{1}^{n}}{R^{n}+K_{1}^{n}}+p_{G3}\cdot\frac{B^{n}}{B^{n}+K_{2}^{n}}-a_{G}G \\ \frac{dS}{dt}=p_{S1}\cdot\frac{AF^{n}}{AF^{n}+K_{3}^{n}}+p_{S2}\cdot\frac{H^{n}}{H^{n}+K_{4}^{n}}-a_{S}S \\ \frac{dAF}{dt}=p_{AF1}\cdot\frac{F^{n}}{F^{n}+K_{5}^{n}}+p_{AF2}\cdot\frac{K_{6}^{n}}{B^{n}+K_{6}^{n}}-a_{AF}AF \\ \frac{dF}{dt}=p_{F1}\cdot\frac{H^{n}}{H^{n}+K_{7}^{n}}+p_{F2}\cdot\frac{AF^{n}}{AF^{n}+K_{8}^{n}}-a_{F}F \\ \frac{dR}{dt}=p_{R}\cdot\frac{K_{9}^{n}}{S^{n}+K_{9}^{n}}-a_{R}R \\ \frac{dB}{dt}=p_{B}\cdot\frac{K_{10}^{n}}{G^{n}+K_{10}^{n}}-a_{B}B \end{matrix}$$

***Late Stage Model description***

In the LSN, later expressed *Hox* genes activate *Grem1* and *Shh*, and are themselves activated by the AER-*Fgf*’s and inhibited by *Gli3R*. *Bmp4* inhibits AER Fgf’s and activates *Grem1*, while *Grem1* inhibits *Bmp4*. Finally, the AER-*Fgf*’s activate *Shh*, and *Shh* inhibits *Gli3R* which in turn inhibits Grem 1 (Fig. 1B). In the LSN, we also include the AER-*Fgf*’s activated Repressor *X* that represses *Grem1* expression.

In our mathematical model of these interactions, *Hox A/D* (H) degrades with first order kinetics (linear degradation at a rate a_H_). It is positively regulated by the AER-*Fgf*’s (AF) and negatively regulated by *Gli3R* (R) according to Hill function kinetics with maximal velocities p_H2_ and p_H1_ and half maximal induction concentrations K_9_ and K_8_ , respectively. The activity of *Grem1* (G) is positively regulated by *Hox A/D* (H) and *Bmp4* (B), inhibited by *Gli3R* (R) and Repressor *X* and degrades with first order kinetics at a rate a_G_. The regulations are modeled with Hill functions with maximal velocities p_G1_, p_G3_, p_G2_, d_G_ and half maximal induction concentrations K_0_, K_2_, K_1_, K_10_, respectively. The activity and concentration of *Shh* (S) is positively controlled by both the AER-*Fgf*’s (AF) and *Hox A/D*. These interactions are modeled by Hill functions with maximal velocity p_S1_, p_S2_ and half maximal induction concentrations K_3,_ K_4_, respectively. The half-life of *Shh* is 1/a_S_. The activity and concentration of the AER-*Fgf*’s (AF) are negatively controlled by *Bmp4* (B) according to a Hill function with maximal velocity p_AF_ and half maximal induction concentration K_5_. The half-life of the AER-*Fgf*’s is 1/a_AF_. The equations governing the rates of change of the activity and concentration of *Gli3R* (R) and *Bmp4* (B) remain the same as in the early stage model, except the maximal induction concentrations are now denoted by K_6_ and K_7_, respectively. A common Hill exponent *n* is used in all equations except that for Repressor *X*. Repressor *X* is activated by the AER-*Fgf*’s (AF) with maximal velocity p_X_ and maximal induction concentrations K_11_. Its half life is 1/a_X_. Its activation is modeled by a Hill function with exponent m. The parameter values are given in Supplementary Table 2. The system of ordinary differential equations that describes the interactions among these genes is the following.

$$\begin{matrix} \frac{dH}{dt}=p_{H1}\cdot\frac{K_{8}^{n}}{R^{n}+K_{8}^{n}}+p_{H2}\cdot\frac{AF^{n}}{AF^{n}+K_{9}^{n}}-a_{H}H \\ \frac{dG}{dt}=p_{G1}\cdot\frac{H^{n}}{H^{n}+K_{0}}+p_{G2}\cdot\frac{K_{1}^{n}}{R^{n}+K_{1}^{n}}+p_{G3}\cdot\frac{B^{n}}{B^{n}+K_{2}^{n}}-d_{G}G\frac{X^{m}}{X^{m}+K_{10}^{m}}-a_{G}G \\ \frac{dS}{dt}=p_{S1}\cdot\frac{AF^{n}}{AF^{n}+K_{3}^{n}}+p_{S2}\cdot\frac{H^{n}}{H^{n}+K_{4}^{n}}-a_{S}S \\ \frac{dAF}{dt}=p_{AF}\cdot\frac{K_{5}^{n}}{B^{n}+K_{5}^{n}}-a_{AF}AF \\ \frac{dR}{dt}=p_{R}\cdot\frac{K_{6}^{n}}{S^{n}+K_{6}^{n}}-a_{R}R \\ \frac{dB}{dt}=p_{B}\cdot\frac{K_{7}^{n}}{G^{n}+K_{7}^{n}}-a_{B}B \\ \frac{dX}{dt}=p_{X}\cdot\frac{AF^{m}}{AF^{m}+K_{11}^{m}}-a_{X}X \end{matrix}$$

**Supplementary Table 2**. Starting parameter values for the early and late stage networks (in parentheses), including the range of parameters used for the sensitivity analyses.

| **Parameter (Early Stage)** | **Units** | **Sensitivity Analysis Range (default values in parentheses)** |
| --- | --- | --- |
| n | dimensionless | 1-6 (3) |
| l | hrs^-1^ | 0.05-0.60 (0.45) |
| a_H_, a_S_, a_G_, a_F_, a_R_, a_B_ | hrs^-1^ | 0.05-0.85 (0.25) |
| a_AF_ | hrs^-1^ | 0.05-0.45 (0.25) |
| p_G1_, p_G2_, p_G3_, p_S1_, p_S2_, p_AF1_, p_AF2_, p_F1_, p_F2_, p_R_, p_B_ |  | 0.05-0.45 (0.25) |
| K_0_ |  | 0-1.2 (0.4) |
| K_1_ |  | 0-1.2 (0.5) |
| K_2_ |  | 0-1.2 (0.3) |
| K_3_ |  | 0-1.2 (0.45) |
| K_4_ |  | 0-1.2 (0.35) |
| K_5_, K_6_, K_7_, K_8_, K_9_, K_10_ |  | 0-1.2 (0.4) |
| **Parameter (Late Stage)** | **Units** | **Sensitivity Analysis Range** |
| n | dimensionless | 1-6 (3) |
| m | dimensionless | 4-8 (6) |
| d_G_ | hrs^-1^ | 10-200 (100) |
| l | hrs^-1^ | 0.0-1.2 (0) |
| a_H_, a_G_, a_S_, a_AF_, a_R_ | hrs^-1^ | 0.05-0.85 (0.25) |
| p_H1_, p_H2_, p_S1_, p_S2_, p_R_, p_B_ |  | 0.05-0.45 (0.25) |
| K_8_, K_9_, |  | 0-1.2 (0.4) |
| p_G1_, p_G3_ |  | 0.05-0.45 (0.20) |
| p_G2_ |  | 0.05-0.45 (0.30) |
| K_0_ |  | 0-1.2 (1.0) |
| K_1_, K_6_,K_7_ |  | 0-1.2 (0.4) |
| K_2_ |  | 0-1.2 (0.3) |
| K_10_ |  | 0-1.2 (0.1) |
| K_3_ |  | 0-1.2 (0.65) |
| K_4_ |  | 0-1.2 (0.7) |
| p_AF_ |  | 0.05-0.75 (0.5) |
| K_5_ |  | 0-1.2 (0.6) |
| a_B_ | hrs^-1^ | 0.05-0.85 (0.125) |
| a_X_ | hrs^-1^ | 0.005-0.85 (0.01) |
| p_X_ |  | 0.0005-0.45 (0.001) |
| K_11_ |  | 0-1.2 (0.3) |

***Among individual variation in gene expression (qPCR)***

*Embryo collection –* All procedures were within guidelines of the University of Illinois at Urbana-Champaign IACUC. Female ICR mice were set up in the late afternoon with a male of the same strain. The next morning the female was examined for the presence of a semen plug in the vagina. By convention, the mating was recorded to take place at 12:00am that day, thus by 12:00pm on the day the plug was found the embryos were at E0.5. At the appropriate stage pregnant females were euthanized by CO_2_ asphyxiation and death was confirmed by cervical dislocation. The uterus was immediately removed and placed in a sterile petri dish in RNase-free PBS and embryos quickly dissected out. Forceps were sprayed down with RNase*AWAY* (Molecular BioProducts #7000) before being used. Embryos were placed into RNA*later* (Qiagen #76106) promptly following dissection and stored overnight at 4°C before being moved to a -20°C freezer.

*Forelimb collection* – Forelimbs from each embryo (8 embryos/litter) were removed with forceps and microsurgical scissors that had been treated with RNase*AWAY*. Dissection was performed in the storage media, RNA*later* to reduce RNA degradation. The two forelimbs from each embryo were pooled. Forelimbs were stored in RNA*later* at -20ºC until RNA purification was carried out. Tissue was stored in RNA*later* for no longer than 3 weeks prior to purification.

*RNA Purification* - The E.Z.N.A. Total RNA Kit I (OMEGA bio-tek #R6834) was used to purify RNA. If RNA*later* froze, samples were incubated at 37ºC until thawed (10 minutes). The animal tissue protocol was followed according to the manual. To homogenize tissues a Tissuemiser (Fisher Scientific) was used. Before and in between each use the homogenizer tip was cleaned with RNase-free ethanol and washed with RNase-free water. DNase I digestion was not performed. RNA was eluted in 40µL DEPC water, concentration measured on a NanoDrop (ND-1000), and stored at -80°C. RNA concentrations ranged from 20-300ng/μL (higher concentration for older stages). Filter tips were used for RNA purification to avoid contamination. Bench-top and pipettes were wiped with RNase*AWAY* prior to beginning the protocol. Most samples had an A_260_/A_280_ ratio ranging from 1.70 to 2.11, a few were above or below this. No further purity testing was done with the RNA (Bioanalyzer or similar).

*cDNA Synthesis* – cDNA was synthesized using the SuperScript III Reverse Transcriptase kit (Invitrogen #18080). When possible, 1µg of total RNA was used for cDNA synthesis. If RNA concentration was low (due to size of forelimbs) 11µL of RNA was used in the reaction. 1µL of 50µM oligo d(T)23 VN (New England BioLabs #S1327S) was combined with 1µL 10mM dNTP Mix (New England BioLabs #N0447S). Reaction was incubated at 65°C for 5 minutes then placed on ice for at least 1 minute. The rest of the reaction was carried out according to the Invitrogen protocol with the exception that RNase inhibitor was not added. Reaction was incubated for 1 hour at 50°C and then stopped with incubation at 75°C for 10 minutes. Samples were centrifuged briefly and concentration measured with a NanoDrop before being stored at -20°C.

*Primers for qPCR* – Primers were designed with NCBI PrimerBLAST or taken from literature. See Supplementary 3 for genes, accession numbers, primer sequence, and literature citations (if appropriate). Primers were synthesized by either Sigma-Aldrich or IDT. Primers were purified by the manufacturers using standard desalting. They were reconstituted in RNase-free water at 100μM and then a stock solution was made with 160μL water and 20μL each of forward and reverse primer. An *in* *silico* test of primer specificity was done using PrimerBLAST and all were specific to their templates at the time of design.

**Supplementary Table 3.** Primers for qPCR.

| **Gene** | **Accession #** | **Sequence** | **Size** | **Citation** |
| --- | --- | --- | --- | --- |
| *Act-b* | NM_007393.3 | F: CCGCGAGCACAGCTTCTTTG  R: CGCAGCGATATCGTCATCCAT | 82 | n/a |
| *Bmp4* | NM_007554.2 | F: TAGTGCCATTCGGAGCGAC  R: AGTCCATGATTCTTGGGAGCC | 88 | n/a |
| *Fgf8* | NM_010205.2 | F: CAACAAGCGCATCAACGC  R: GAACTCGGACTCTGCTTCCAA | 92 | n/a |
| *Fgf10* | NM_008002.4 | F:CCGTACAGTGTCCTGGAGATAACAT  R: CATGGCTAAGTAATAGTTGCTCTTGAT | 84 | (8) |
| *Gli3* | NM_008130 | F: CTCCCATTCCCAATCCCTATC  R: GGGAGGTCAGCAAAGAACTCAT | 74 | (9) |
| *Grem1* | NM_011824.4 | F: AAGTGACAGAATGAATCGCACC  R: GGACTGGGTCTGCTCAGAGT | 157 | n/a |
| *Shh* | NM_009170.3 | F: ACCCAACTCCGATGTGTTCCGTTA  R: TATAACCTTGCCTGCTGTTGCTGC | 110 | (10) |

*qPCR Reaction Conditions* – The *Power* SYBR Green PCR Master Mix (Applied Biosystems #4367659) was used for qPCR. It contains everything except primers. A reaction volume of 15µL was set up (7.5µL Master Mix, 2µL water, 0.5µL primer mix (forward and reverse combined), and 5µL 4ng/µL cDNA). Reactions were mixed at room temperature and filter tips were used for all samples. Samples were set up in triplicate in 384-well clear plates from Applied Biosystems (#4309849) and covered with Optical Adhesive Covers (Applied Biosystems #4360954). The Applied Biosystems 7900HT Fast RealTime PCR System was used to analyze samples using the Standard Curve method. The standard curve was a series of 1:5 dilutions that were done with every plate for each gene analyzed. The cDNA from the standard curve was purified from the combined forelimbs and hindlimbs of an E10.5 mouse. The default reaction conditions of 40 cycles of 15s at 95.0°C and 1 min at 60.0°C was done, followed by a dissociation curve each time. Due to the number of samples and space limitations on the plate, the 72 total samples analyzed were spread over two to three plates, with a standard curve set up from the same cDNA stocks on each plate.

*Data Analysis* – SDS 2.4.1 (Applied Biosystems) was used to analyze the data. Outliers were removed and any wells that failed to amplify were also omitted. Data were exported to Excel, and quantities for target genes were normalized to the reference gene (beta-actin) by dividing the mean reading for the target (out of 3 replicates) by the mean reading for the reference gene. The slopes of the standard curves for *beta-actin* were consistently between -3.3 to -3.4 and an R^2^ of 0.99 for all runs. The target genes were more variable: for *Bmp4* m=-2.93 to -3.2 and R^2^ = 0.984 to 0.991, for *Fgf8* m=-3.0 to -3.2, R^2^ = 0.979 to 0.988, for *Fgf10* m=-2.97 to -3.4 and R^2^ = 0.984 to 0.991, for *Shh* m=-2.73 to -3.67 and R^2^ = 0.982 to 0.991, and for *Grem1* m=-2.79 to -3.57 and R^2^ = 0/971 to 0.989. For all genes the dissociation curve was consistent with a single product.

***Gene expression variation among species (RNASeq)*** – Embryonic mice and opossums were obtained from timed matings in the breeding colonies housed in the Sears Lab at the University of Illinois. Pig embryos were obtained through timed ovulations following inseminations at the University of Illinois pig farm. Bat embryos were obtained from field collections in Trinidad. Limbs were removed from these embryos and used to perform RNASeq analysis.

Resulting RNASeq sequences were preprocessed to remove Illumina adaptors and bases with qualities below 20 at the read's end were trimmed. The preprocessed libraries where then aligned to their corresponding reference genomes. For mouse, opossum, and pig, we used the Ensembl reference genomes and annotation files corresponding to assemblies, GRCm38, BROADO5, and Sscrofa10.2, respectively. The bat species we are using (*Carollia perspicillata*) does not have a published genome. We initially attempted to align the processed reads against *Myotis lucifugus*, a different bat species with an available genome sequence and gene annotation in ENSEMBL. However, the mapping (using Tophat) resulted in only ~9% alignment rates. For this reason, we resorted to a de novo transcriptome assembly strategy for our bat data. To do this, we pooled all bat libraries and fed them into Trinity (1), a de-novo transcript assembly tool. Trinity predicted 350,733 gene isoforms which were filtered to keep only those matching (blastx, E-value < 1e-20) (2) the protein sequences of the SwissProt database (3). This resulted in 88,930 gene sequences. These sequences were used as references for the mapping of  the RNA-seq libraries (RSEM, which internally uses bowtie). With this *de novo* assembly, the bat alignment rates improved by an order of magnitude, being, on average, 90%. This enormous improvement in mappability convinced us to use this *de novo* transcriptome for all our bat analyses.

For the species with a reference genome (mouse, opossum, and pig) we aligned the reads using STAR (4) and then used Cufflinks (5) to compute their gene expression. For bat, read alignment and gene expression were computed with RSEM (6) using as reference sequences the 57,349 gene isoforms previously described. For all four species, gene expression values were normalized as fragments per kilobase of transcript per million mapped reads (FPKM), and to account for differences in the mass composition between samples we performed normalization across corresponding samples as described by Anders et al., 2010 (7).

We analyzed the conservation of the gene expression profiles of bat, mouse, opossum, and pig across embryonic limb development using the mean of all species pairwise Spearman coefficients ( c ):

Where r i , j is the Spearman coefficient between species i and j at a given stage, and

k is the total number of species under study. We used the Spearman rather than Pearson coefficient, as the former is more robust against outliers.

References

1. Grabherr MG*, et al.* (2011) Full-length transcriptome assembly from RNA-Seq data without a reference genome. *Nat Biotechnol* 29(7):644-652.

2. Camacho C, *et al.* (2009) BLAST+: Architecture and applications. *BMC Bioinformatics* 10:421.

3. Bairoch A & Apweiler R (1997) The SWISS-PROT protein sequence data bank and its supplement TrEMBL. *Nucleic Acids Res* 25(1):31-36.

4. Dobin A, *et al.* (2013) STAR: ultrafast universal RNA-seq aligner. *Bioinformatics* 29:15-21.

5. Trapnell C*, et al.* (2010) Transcript assembly and quantification by RNA-Seq reveals unannotated transcripts and isoform switching during cell differentiation. *Nat Biotechnol* 28(5):511-515.

6. Li B & Dewey CN (2011) RSEM: accurate transcript quantification from RNA-Seq data with or without a reference genome. *BMC Bioinformatics* 12:323.

7. Anders S & Huber W (2010) Differential expression analysis for sequence count data. *Genome Biol* 11:R106.

8. Kruger C & Kappen C (2010) Expression of cartilage developmental genes in *Hoxc8*- and *Hoxd4*- transgenic mice. *PLoS One* 5:e8978.

9. Renault MA (2010) *Sonic hedgehog* induces angiogensis via Rho kinase-dependent signaling in endothelial cells. *J Mol Cell Cardiol* 49:490-498.

10. Desouza LA, *et al.* (2011) Thyroid hormone regulates the expression of the *Sonic hedghog* signaling pathway in the embryonic and adult mammalian brain. *Endocrinology* 152:1989-2000.
