# Supplementary figures and images for: The Relationship between Gene Network Structure and Expression Variation among Individuals and Species
Source: PLoS Genet. 2015 Aug 28;11(8):e1005398. doi: 10.1371/journal.pgen.1005398 (PMC4552942; doi:10.1371/journal.pgen.1005398)

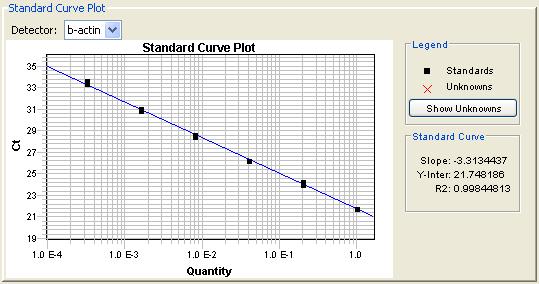

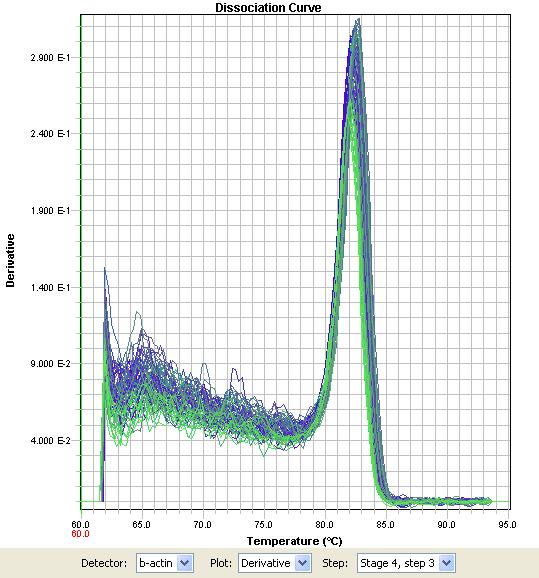


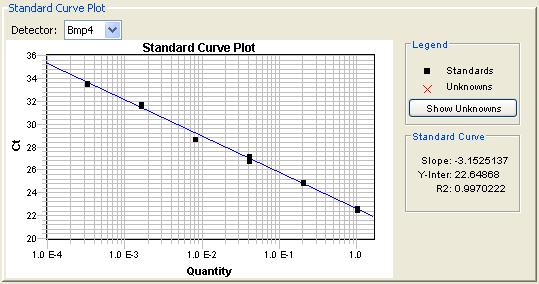


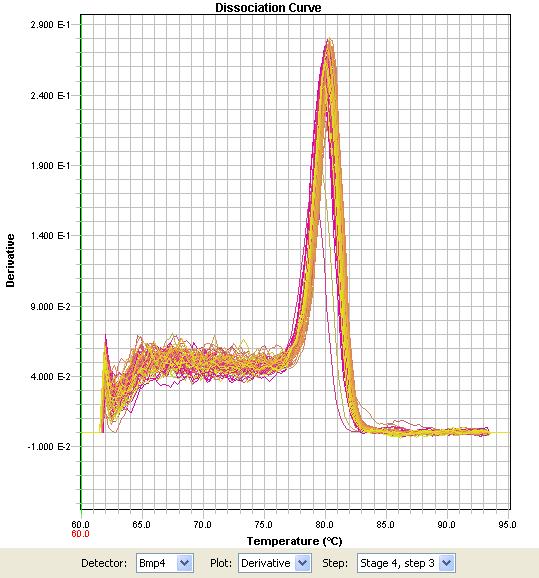


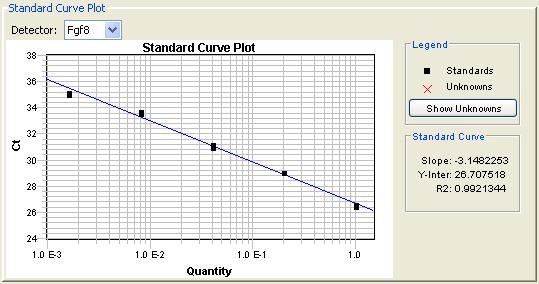


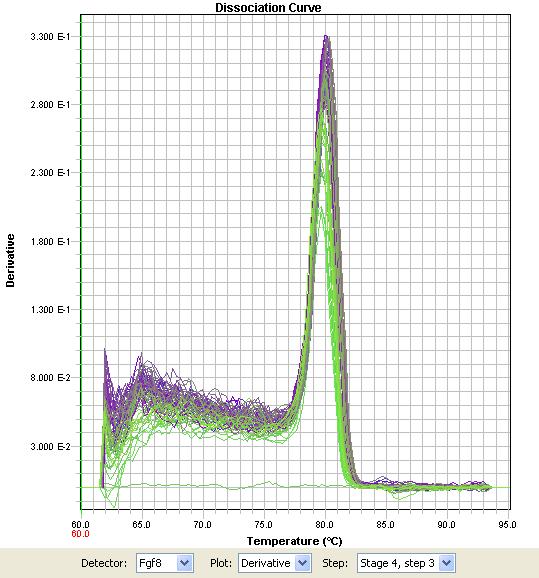


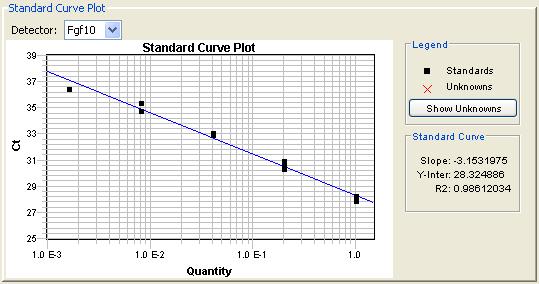


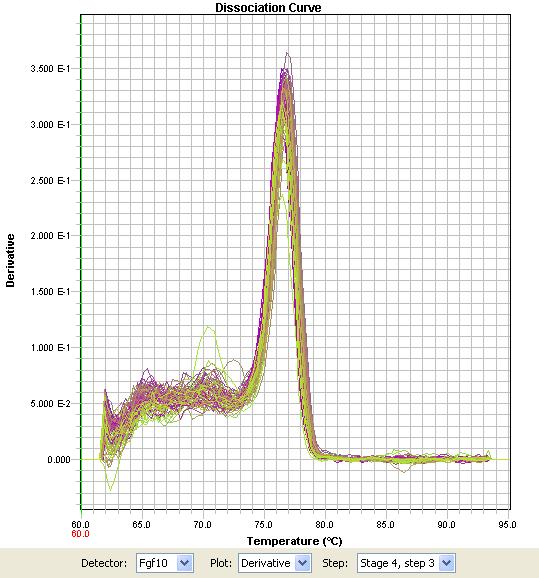


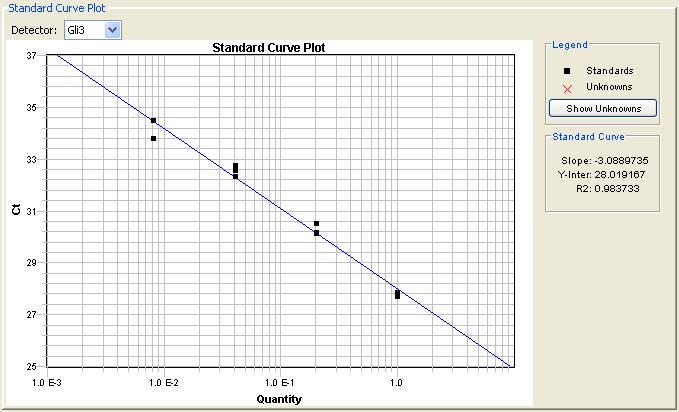


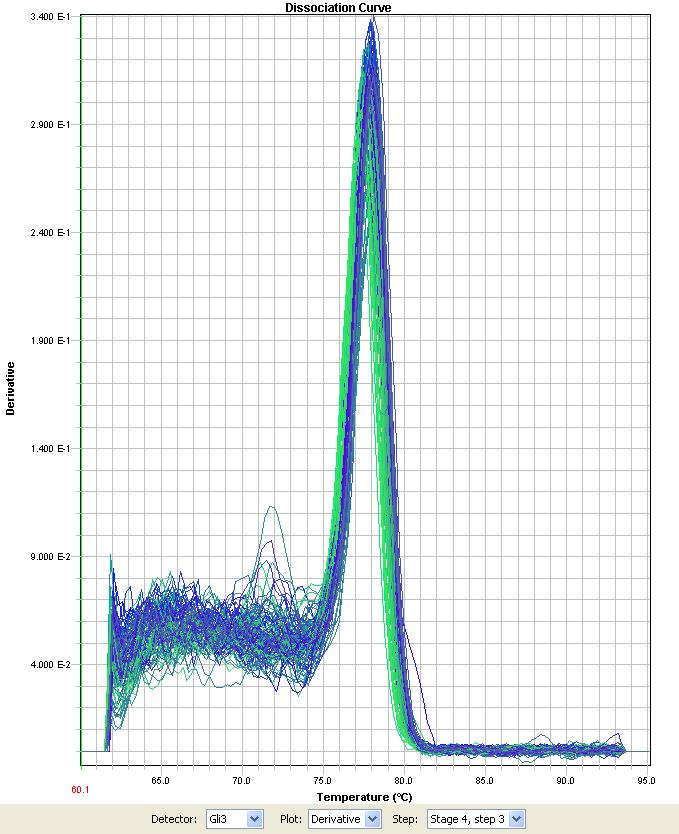


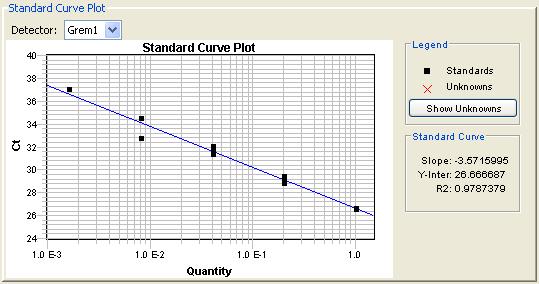


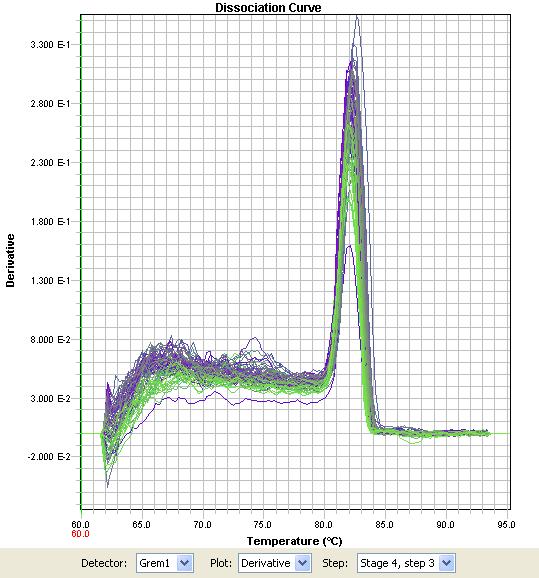


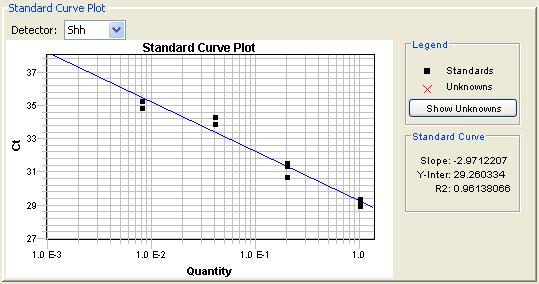


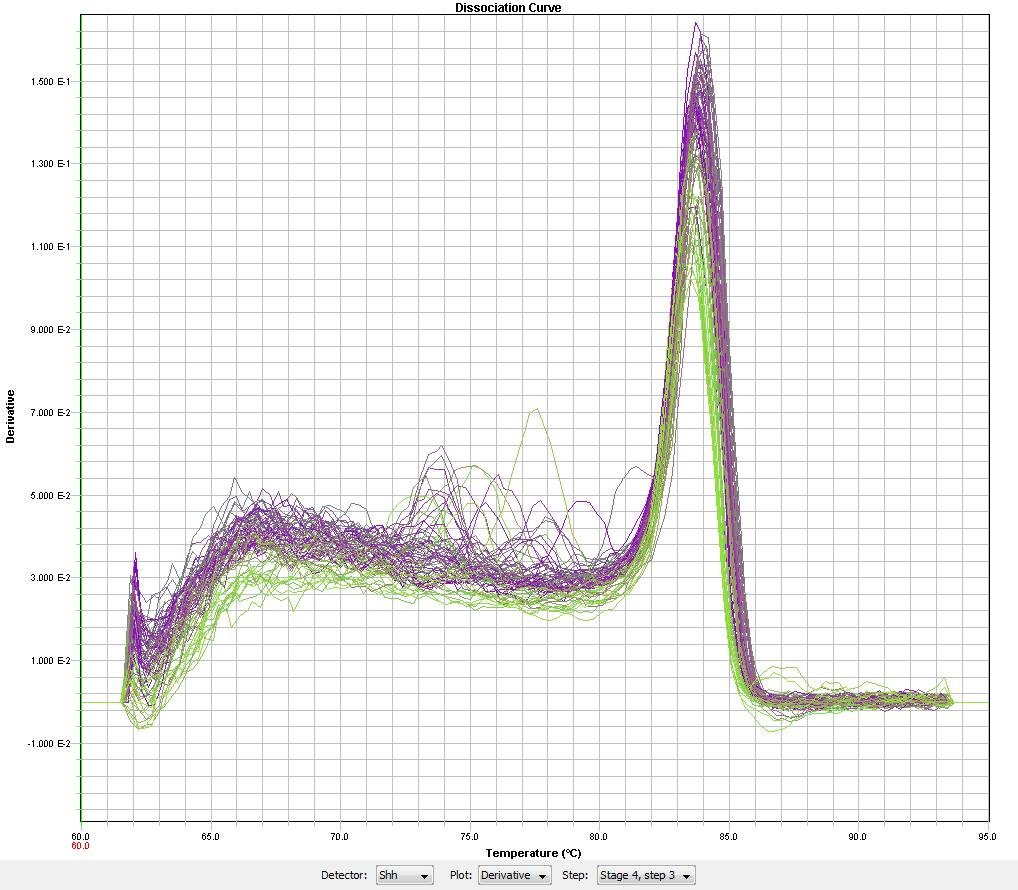

Supplement: S2 Methods — (DOCX) [file pgen.1005398.s003.docx]
